# Intraoperative ventilator settings and their association with postoperative pulmonary complications in neurosurgical patients: post-hoc analysis of LAS VEGAS study

**DOI:** 10.1186/s12871-020-00988-x

**Published:** 2020-04-02

**Authors:** Chiara Robba, Sabrine N. T. Hemmes, Ary Serpa Neto, Thomas Bluth, Jaume Canet, Michael Hiesmayr, M. Wiersma Hollmann, Gary H. Mills, Marcos F. Vidal Melo, Christian Putensen, Samir Jaber, Werner Schmid, Paolo Severgnini, Hermann Wrigge, Denise Battaglini, Lorenzo Ball, Marcelo Gama de Abreu, Marcus J. Schultz, Paolo Pelosi, FERS for the LAS VEGAS investigators, Wolfgang Kroell, Helfried Metzler, Gerd Struber, Thomas Wegscheider, Hans Gombotz, Michael Hiesmayr, Werner Schmid, Bernhard Urbanek, David Kahn, Mona Momeni, Audrey Pospiech, Fernande Lois, Patrice Forget, Irina Grosu, Jan Poelaert, Veerle van Mossevelde, Marie-Claire van Malderen, Dimitri Dylst, Jeroen van Melkebeek, Maud Beran, Stefan de Hert, Luc De Baerdemaeker, Bjorn Heyse, Jurgen Van Limmen, Piet Wyffels, Tom Jacobs, Nathalie Roels, Ann De Bruyne, Stijn van de Velde, Brigitte Leva, Sandrine Damster, Benoit Plichon, Marina Juros-Zovko, Dejana Djonoviċ-Omanoviċ, Selma Pernar, Josip Zunic, Petar Miskovic, Antonio Zilic, Slavica Kvolik, Dubravka Ivic, Darija Azenic-Venzera, Sonja Skiljic, Hrvoje Vinkovic, Ivana Oputric, Kazimir Juricic, Vedran Frkovic, Jasminka Kopic, Ivan Mirkovic, Nenad Karanovic, Mladen Carev, Natasa Dropulic, Jadranka Pavicic Saric, Gorjana Erceg, Matea Bogdanovic Dvorscak, Branka Mazul-Sunko, Anna Marija Pavicic, Tanja Goranovic, Branka Maldini, Tomislav Radocaj, Zeljka Gavranovic, Inga Mladic-Batinica, Mirna Sehovic, Petr Stourac, Hana Harazim, Olga Smekalova, Martina Kosinova, Tomas Kolacek, Kamil Hudacek, Michal Drab, Jan Brujevic, Katerina Vitkova, Katerina Jirmanova, Ivana Volfova, Paula Dzurnakova, Katarina Liskova, Radovan Dudas, Radek Filipsky, Samir el Kafrawy, Hisham Hosny Abdelwahab, Tarek Metwally, Ahmed Abdel-Razek, Ahmed Mostafa El-Shaarawy, Wael Fathy Hasan, Ahmed Gouda Ahmed, Hany Yassin, Mohamed Magdy, Mahdy Abdelhady, Mohamed Mahran, Eiko Herodes, Peeter Kivik, Juri Oganjan, Annika Aun, Alar Sormus, Kaili Sarapuu, Merilin Mall, Juri Karjagin, Emmanuel Futier, Antoine Petit, Adeline Gerard, Emmanuel Marret, Marc Solier, Samir Jaber, Albert Prades, Jens Krassler, Simone Merzky, Marcel Gama de Abreu, Christopher Uhlig, Thomas Kiss, Anette Bundy, Thomas Bluth, Andreas Gueldner, Peter Spieth, Martin Scharffenberg, Denny Tran Thiem, Thea Koch, Tanja Treschan, Maximilian Schaefer, Bea Bastin, Johann Geib, Martin Weiss, Peter Kienbaum, Benedikt Pannen, Andre Gottschalk, Mirja Konrad, Diana Westerheide, Ben Schwerdtfeger, Hermann Wrigge, Philipp Simon, Andreas Reske, Christian Nestler, Dimitrios Valsamidis, Konstantinos Stroumpoulis, Georgios Antholopoulos, Antonis Andreou, Dimitris Karapanos, Kassiani Theodoraki, Georgios Gkiokas, Marios-Konstantinos Tasoulis, Tatiana Sidiropoulou, Foteini Zafeiropoulou, Panagiota Florou, Aggeliki Pandazi, Georgia Tsaousi, Christos Nouris, Chryssa Pourzitaki, Dmitri Bystritski, Reuven Pizov, Arieh Eden, Caterina Valeria Pesce, Annamaria Campanile, Antonella Marrella, Salvatore Grasso, Michele De Michele, Francesco Bona, Gianmarco Giacoletto, Elena Sardo, Luigi Giancarlo, Vicari Sottosanti, Maurizio Solca, Carlo Alberto Volta, Savino Spadaro, Marco Verri, Riccardo Ragazzi, Roberto Zoppellari, Gilda Cinnella, Pasquale Raimondo, Daniela La Bella, Lucia Mirabella, Davide D’antini, Paolo Pelosi, Alexandre Molin, Iole Brunetti, Angelo Gratarola, Giulia Pellerano, Rosanna Sileo, Stefano Pezzatto, Luca Montagnani, Laura Pasin, Giovanni Landoni, Alberto Zangrillo, Luigi Beretta, Ambra Licia Di Parma, Valentina Tarzia, Roberto Dossi, Marta Eugenia Sassone, Daniele Sances, Stefano Tredici, Gianluca Spano, Gianluca Castellani, Luigi Delunas, Sopio Peradze, Marco Venturino, Ines Arpino, Sara Sher, Concezione Tommasino, Francesca Rapido, Paola Morelli, Maria Vargas, Giuseppe Servillo, Andrea Cortegiani, Santi Maurizio Raineri, Francesca Montalto, Vincenzo Russotto, Antonino Giarratano, Marco Baciarello, Michela Generali, Giorgia Cerati, Yigal Leykin, Filippo Bressan, Vittoria Bartolini, Lucia Zamidei, Luca Brazzi, Corrado Liperi, Gabriele Sales, Laura Pistidda, Paolo Severgnini, Elisa Brugnoni, Giuseppe Musella, Alessandro Bacuzzi, Dalip Muhardri, Agreta Gecaj-Gashi, Fatos Sada, Adem Bytyqi, Aurika Karbonskiene, Ruta Aukstakalniene, Zivile Teberaite, Erika Salciute, Renatas Tikuisis, Povilas Miliauskas, Sipylaite Jurate, Egle Kontrimaviciute, Gabija Tomkute, John Xuereb, Maureen Bezzina, Francis Joseph Borg, Sabrine Hemmes, Marcus Schultz, Markus Hollmann, Irene Wiersma, Jan Binnekade, Lieuwe Bos, Christa Boer, Anne Duvekot, Bas in ’t Veld, Alice Werger, Paul Dennesen, Charlotte Severijns, Jasper De Jong, Jens Hering, Rienk van Beek, Stefan Ivars, Ib Jammer, Alena Breidablik, Katharina Skirstad Hodt, Frode Fjellanger, Manuel Vico Avalos, Jannicke Mellin-Olsen, Elisabeth Andersson, Amir Shafi-Kabiri, Ruby Molina, Stanley Wutai, Erick Morais, Glória Tareco, Daniel Ferreira, Joana Amaral, Maria de Lurdes Goncalves Castro, Susana Cadilha, Sofia Appleton, Suzana Parente, Mariana Correia, Diogo Martins, Angela Monteirosa, Ana Ricardo, Sara Rodrigues, Lucian Horhota, Ioana Marina Grintescu, Liliana Mirea, Ioana Cristina Grintescu, Dan Corneci, Silvius Negoita, Madalina Dutu, Ioana Popescu Garotescu, Daniela Filipescu, Alexandru Bogdan Prodan, Gabriela Droc, Ruxandra Fota, Mihai Popescu, Dana Tomescu, Ana Maria Petcu, Marian Irinel Tudoroiu, Alida Moise, Catalin-Traian Guran, Iorel Gherghina, Dan Costea, Iulia Cindea, Sanda-Maria Copotoiu, Ruxandra Copotoiu, Victoria Barsan, Zsolt Tolcser, Magda Riciu, Septimiu Gheorghe Moldovan, Mihaly Veres, Alexey Gritsan, Tatyana Kapkan, Galina Gritsan, Oleg Korolkov, Alexander Kulikov, Andrey Lubnin, Alexey Ovezov, Pavel Prokoshev, Alexander Lugovoy, Natalia Anipchenko, Andrey Babayants, Irina Komissarova, Karginova Zalina, Valery Likhvantsev, Sergei Fedorov, Aleksandra Lazukic, Jasmina Pejakovic, Dunja Mihajlovic, Zuzana Kusnierikova, Maria Zelinkova, Katarina Bruncakova, Lenka Polakovicova, Villiam Sobona, Barbka Novak-Supe, Ana Pekle-Golez, Miroljub Jovanov, Branka Strazisar, Jasmina Markovic-Bozic, Vesna Novak-Jankovic, Minca Voje, Andriy Grynyuk, Ivan Kostadinov, Alenka Spindler-Vesel, Victoria Moral, Mari Carmen Unzueta, Carlos Puigbo, Josep Fava, Jaume Canet, Enrique Moret, Mónica Rodriguez Nunez, Mar Sendra, Andrea Brunelli, Frederic Rodenas, Pablo Monedero, Francisco Hidalgo Martinez, Maria Jose Yepes Temino, Antonio Martínez Simon, Ana de Abajo Larriba, Alberto Lisi, Gisela Perez, Raquel Martinez, Manuel Granell, Jose Tatay Vivo, Cristina Saiz Ruiz, Jose Antonio de Andrés Ibañez, Ernesto Pastor, Marina Soro, Carlos Ferrando, Mario Defez, Cesar Aldecoa Alvares-Santullano, Rocio Perez, Jesus Rico, Monir Jawad, Yousif Saeed, Lars Gillberg, Zuleyha Kazak Bengisun, Baturay Kansu Kazbek, Nesil Coskunfirat, Neval Boztug, Suat Sanli, Murat Yilmaz, Necmiye Hadimioglu, Nuzhet Mert Senturk, Emre Camci, Semra Kucukgoncu, Zerrin Sungur, Nukhet Sivrikoz, Serpil Ustalar Ozgen, Fevzi Toraman, Onur Selvi, Ozgur Senturk, Mine Yildiz, Bahar Kuvaki, Ferim Gunenc, Semih Kucukguclu, Şule Ozbilgin, Jale Maral, Seyda Canli, Oguzhan Arun, Ali Saltali, Eyup Aydogan, Fatma Nur Akgun, Ceren Sanlikarip, Fatma Mine Karaman, Andriy Mazur, Sergiy Vorotyntsev, Guy Rousseau, Colin Barrett, Lucia Stancombe, Ben Shelley, Helen Scholes, James Limb, Amir Rafi, Lisa Wayman, Jill Deane, David Rogerson, John Williams, Susan Yates, Elaine Rogers, Mark Pulletz, Sarah Moreton, Stephanie Jones, Suresh Venkatesh, Maudrian Burton, Lucy Brown, Cait Goodall, Matthew Rucklidge, Debbie Fuller, Maria Nadolski, Sandeep Kusre, Michael Lundberg, Lynn Everett, Helen Nutt, Maka Zuleika, Peter Carvalho, Deborah Clements, Ben Creagh-Brown, Philip Watt, Parizade Raymode, Rupert Pearse, Otto Mohr, Ashok Raj, Thais Creary, Ahmed Chishti, Andrea Bell, Charley Higham, Alistair Cain, Sarah Gibb, Stephen Mowat, Danielle Franklin, Claire West, Gary Minto, Nicholas Boyd, Gary Mills, Emily Calton, Rachel Walker, Felicity Mackenzie, Branwen Ellison, Helen Roberts, Moses Chikungwa, Clare Jackson, Andrew Donovan, Jayne Foot, Elizabeth Homan, Jane Montgomery, David Portch, Pauline Mercer, Janet Palmer, Jonathan Paddle, Anna Fouracres, Amanda Datson, Alyson Andrew, Leanne Welch, Alastair Rose, Sandeep Varma, Karen Simeson, Mrutyunjaya Rambhatla, Jaysimha Susarla, Sudhakar Marri, Krishnan Kodaganallur, Ashok Das, Shivarajan Algarsamy, Julie Colley, Simon Davies, Margaret Szewczyk, Thomas Smith, Ana Fernandez-Bustamante, Elizabeth Luzier, Angela Almagro, Marcos Vidal Melo, Luiz Fernando, Demet Sulemanji, Juraj Sprung, Toby Weingarten, Daryl Kor, Federica Scavonetto, Yeo Tze, the PROtective VEntilation Network and the Clinical Trial Network of the European Society of Anaesthesiology

**Affiliations:** 1 Anaesthesia and Intensive Care, San Martino Policlinico Hospital, IRCCS for Oncology and Neurosciences, Largo Rosanna Benzi 8, 16131 Genoa, Italy; 2 Department of Intensive Care, Amsterdam University Medical Centers, location ‘AMC’, Amsterdam, The Netherlands; 3 Department of Anaesthesiology, Amsterdam University Medical Centers, location ‘AMC’, Amsterdam, The Netherlands; 4 Department of Critical Care Medicine, Hospital Israelita Albert Einstein, Sao Paulo, Brazil; 5 Department of Anaesthesiology and Intensive Care Medicine, Pulmonary engineering group, University Hospital Carl Gustav Carus, Technische Universitat Dresden, Dresden, Germany; 6 Department of Anaesthesiology and Postoperative Care, Hospital Universitari Germans Trials I Pujol, Barcelona, Spain; 7 Division Cardiac, Thoracic, Vascular Anesthesia and Intensive Care, Medical University Vienna, Vienna, Austria; 8 Operating Services, Critical Care and Anaesthesia, Sheffield Teaching Hospitals and University of Sheffield, Sheffield, UK; 9 Department of Anaesthesia, Critical Care and Pain Medicine, Massachussetts General Hospital, Boston, MA USA; 10 Department of Anesthesiology and Intenisve Care Medicine, University Hospital Bonn, Bonn, Germany; 11 Department of Anaesthesia and Intensive Care, Saint Eloi Montpellier University Hospital, and PhyMedExp, University of Montpellier, Montpellier, France; 12 Department of Biotechnology and Sciences of Life, ASST-Setteleghi Ospedale di circolo e Fondazione Macchi, University of Insubria, Varese, Italy; 13 Department of Anesthesiology and Intensive Care Medicine, University of Leipzig, Leipzig, Germany; 14 Department of Surgical Sciences and Integrated Diagnostics, University of Genoa, Genoa, Italy; 15 Mahidol-Oxford Tropical Medicine Research Unit (MORU), Mahidol University, Bangkok, Thailand

**Keywords:** LAS VEGAS, Mechanical ventilation, Postoperative pulmonary complications, Neurosurgery

## Abstract

**Background:**

Limited information is available regarding intraoperative ventilator settings and the incidence of postoperative pulmonary complications (PPCs) in patients undergoing neurosurgical procedures. The aim of this post-hoc analysis of the ‘Multicentre Local ASsessment of VEntilatory management during General Anaesthesia for Surgery’ (LAS VEGAS) study was to examine the ventilator settings of patients undergoing neurosurgical procedures, and to explore the association between perioperative variables and the development of PPCs in neurosurgical patients.

**Methods:**

Post-hoc analysis of LAS VEGAS study, restricted to patients undergoing neurosurgery. Patients were stratified into groups based on the type of surgery (brain and spine), the occurrence of PPCs and the assess respiratory risk in surgical patients in Catalonia (ARISCAT) score risk for PPCs.

**Results:**

Seven hundred eighty-four patients were included in the analysis; 408 patients (52%) underwent spine surgery and 376 patients (48%) brain surgery. Median tidal volume (V_T_) was 8 ml [Interquartile Range, IQR = 7.3–9] per predicted body weight; median positive end–expiratory pressure (PEEP) was 5 [3 to 5] cmH_2_0. Planned recruitment manoeuvres were used in the 6.9% of patients. No differences in ventilator settings were found among the sub-groups. PPCs occurred in 81 patients (10.3%). Duration of anaesthesia (odds ratio, 1.295 [95% confidence interval 1.067 to 1.572]; *p* = 0.009) and higher age for the brain group (odds ratio, 0.000 [0.000 to 0.189]; *p* = 0.031), but not intraoperative ventilator settings were independently associated with development of PPCs.

**Conclusions:**

Neurosurgical patients are ventilated with low V_T_ and low PEEP, while recruitment manoeuvres are seldom applied. Intraoperative ventilator settings are not associated with PPCs.

## Background

Lung–protective ventilation strategies are increasingly used in surgical patients [1, 2]. Typical lung–protective strategies include the use of a low tidal volume (V_T_) and a low plateau pressure (Pplat), with moderate positive end–expiratory pressure (PEEP) and use of recruitment manoeuvres (RM) if needed [1, 2]. Among these settings, a low V_T_ seems to have the most protective effects compared with moderate or high PEEP [3, 4].

However, lung–protective ventilation is rarely used in brain injured patients, in whom median V_T_ is generally 9 ml/kg of predicted body weight (PBW) [5]. The role of intraoperative ventilator settings and their potential impacts on the development of postoperative complications (PPCs) has been scarcely evaluated in neurological patients [6]. Typically, patients with neurosurgical pathologies have been excluded from most trials on protective intraoperative ventilation. This may be because lung–protective strategies could have detrimental effects on cerebrovascular physiology, and thus might be potentially contraindicated in acute neurosurgical patients [7]. Moreover, just few and inconclusive data exist regarding the ventilator settings applied in patients undergoing spinal surgery and the incidence of PPCs in this population [8, 9].

We therefore conducted a post-hoc analysis of the ‘Local ASsessment ofVEntilatory management during General Anaesthesia for Surgery–study’ (LAS VEGAS), a conveniently sized international observational study in the operating rooms of patients receiving mechanical ventilation [10]. We focused on neurosurgical patients, including patients undergoing brain or spine surgery. The aims of this analysis were to assess which ventilator strategies were used in neurosurgical patients during general anaesthesia, and to assess the incidence of PPCs and risk factors (including type of surgery, ventilator settings, risk for PPCs) associated with the development of PPCs. The main hypothesis tested was that neurosurgical patients are ventilated with high tidal volume and low positive end expiratory pressure, and that intraoperative ventilator settings can have an effect on PPCs development.

## Methods

### LAS VEGAS study

This article is reported as per Strengthening the Reporting of Observational Studies in Epidemiology (STROBE) reporting guidelines (www.strobe-statemenent.org) (Electronic supplementary material ESM Table S1).

LAS VEGAS [8] was an international multicentre observational prospective study (registered at www. clinicaltrials.gov (study identifier NCT01601223)), endorsed and supported by the European Society of Anaesthesiology and the Amsterdam University Medical Centres, location AMC, Amsterdam, The Netherlands. Details about the LAS VEGAS study collaborators, participating centres and hospital characteristics of participating centres are reported in ESM Tables S2a, b and S3.

All adult patients requiring invasive ventilation for surgical procedures in a time window of 7 days were included. Exclusion criteria were: age under 18 years, obstetric procedures, recent ventilation before surgery (< 28 days), surgical procedures not performed in the operating room, and interventions requiring cardiopulmonary bypass.

For this study, we restricted the analysis to patients receiving intraoperative ventilation for neurosurgical procedures (brain or spine surgery) (ESM Flow Chart).

### Data collection

After inclusion, the following data were collected: patients’ baseline and demographic characteristics; the assess respiratory risk in surgical patients in Catalonia (ARISCAT) score [11]; American Society of Anaesthesiologists (ASA) scale; details on the surgical procedure including intraoperative hourly vital parameters and ventilation data (mode of ventilation, fraction of inspired oxygen (FiO_2_), V_T_, PEEP, peak pressure (Ppeak), respiratory rate (RR)), end-tidal CO_2_ (ETCO_2_), oxygen saturation (SpO_2_), number and type of recruitment manoeuvres, and intraoperative complications.

Recruitment manoeuvres were defined as ‘rescue’ when the recruitment manoeuvre was not part of the planned ventilation strategy and defined as ‘planned’ if it was part of routine ventilation practice (ESM Table S4). Mechanical power (MP) was calculated according to the following formula [12]: 0.098 x V_T_ x RR x [Ppeak x (Pplat - PEEP)/2]. Hourly data were collected starting at the induction of anaesthesia (T1/40) and then hourly until the end of anaesthesia, up to the 7th hour of surgery (T1/47).

### Endpoints

The primary endpoint was to describe the current practice and ventilator strategies in patients undergoing neurosurgical interventions, in particular ventilator mode, V_T_, PEEP, driving pressure, Ppeak and Pplat and RR, as well as mechanical power.

The secondary outcome was to assess the prevalence of PPCs and the association with preoperative and intraoperative variables including mechanical ventilator settings, type of surgery, ARISCAT score. Detailed definitions of the composites of PPCs and severe PPCs are provided in ESM Table S5. Intraoperative complications included desaturation, rescue recruitment manoeuvres, need for airway pressure reduction, expiratory flow limitation, hypotension and use of vasoactive drugs, onset of a new cardiac arrhythmia (ESM Table S6). The occurrence of each type of PPC was monitored until hospital discharge, but maximum up to postoperative day 5.

Other secondary endpoints included the occurrence of severe PPCs, intraoperative complications, in-hospital mortality and length of hospital stay.

#### Statistical analysis

Patients were stratified into groups based on type of surgery (brain and spine), the occurrence of PPCs and risk for PPC according to ARISCAT (low risk [ARISCAT < 26] vs. moderate-to-high risk [ARISCAT ≥26]. Continuous variables are expressed as mean ± standard deviation (SD) or median (interquartile range [IQR]) per variable distribution. Discrete variables are presented as percentages. Baseline characteristics among type of neurosurgery were compared by either t-test, Wilcoxon rank-sum test, or chi-squared tests, as appropriate. The effect of type of neurosurgery on the incidence (per 10 P-days) of in-hospital PPC, severe PPC, and discharged alive was evaluated using log-rank test (stratified by centre); differences in survival probabilities and hospital discharge were depicted with an outcome-specific Kaplan-Meier plot.

A multivariable regression model was built, with PPC as dependent variables. Because this outcome is binary (0/1), a logistic regression analysis was applied. Candidate covariates were chosen based on previous medical knowledge, independent of their *p*-value. From this preliminary selection, those variables with *P* < 0.20 in the univariate analysis were preferentially chosen for the stepwise procedure. Then, a reduced and parsimonious model was derived using backward stepwise selection. During this selection process, the linearity assumption for continuous variables was tested and transformed, if appropriate, with fractional polynomials (14). In all regression models, the Huber/White/sandwich estimator of variance correction was applied to account for any clustering effect due to centre sampling.

We set a two-sided *p* value of < 0.05 as the threshold for statistical significance. Stata 15.1 (Stata Statistical Software, release 15 [2017] (Stata Corp LP, College Station, TX, USA), and R (Version 3.5.3; R Foundation for Statistical Computing, Vienna, Austria) were used.

## Results

A total of 784 patients were included in the analysis. Of these, 408 (52%) underwent spine surgery and 376(48%) brain surgery. The characteristics of the patients according to subgroups are described in Table 1. Patients with moderate-to-high risk for PPCs- compared to those at low risk were older, with a higher incidence of co-morbidities (in particular chronic kidney failure), worse ASA physical status, and worse pre-hospital functional status and preoperative conditions (as for laboratory tests and vital signs) (Table 1). Patients who developed PPCs were older, with more frequent co-morbidities (in particular respiratory and cardiological), worse ASA and preoperative functional status (Table 1).

**Table 1:** Pre-Operative Characteristics of the Patients According to Subgroups

	All Patients	Brain	Spine	***p*** value	All patients	PPC	No PPC	***p*** value	All patients	ARISCAT < 26	ARISCAT ≥ 26	***p*** value
***n (%)***	784 (100)	376 (48)	408 (52)		777 (100.0)	81 (10.4)	696 (89.6)		548 (73.3)	200 (26.7)	748 (100.0)	
**Demographics**												
Age, years, mean (SD)	53 (16)	52 (16)	54 (15)	0.104	53 (16)	59 (15)	52 (16)	0.000	50 (15)	51 (15)	63 (16)	0.000
Gender, n (%)												
Male	392 (50)	183 (48.7)	209 (51.2)	0.060	392 (50.5)	37 (45.7)	355 (51.0)	0.072	285 (52.0)	97 (48.5)	382 (51.1)	0.065
Ethnicity, n (%)												
Black	2 (0.3)	0 (0.0)	2 (0.5)		2 (0.3)	2 (2.5)	0 (0.0)		1 (0.2)	1 (0.5)	2 (0.3)	0.663
Caucasian	709 (90.4)	340 (90.4)	369 (90.4)		704 (90.6)	71 (87.7)	633 (90.9)		493 (90.0)	183 (91.5)	676 (90.4)	
Asian	4 (0.5)	1 (0.3)	3 (0.7)		4 (0.5)	1 (1.2)	3 (0.4)		4 (0.7)	0 (0.0)	4 (0.5)	
Other	33 (4.2)	13 (3.5)	20 (4.9)		33 (4.2)	2 (2.5)	31 (4.5)		26 (4.7)	6 (3.0)	32 (4.3)	
**Anthropometry**												
Height, cm, mean (SD)	170 (10)	170 (10)	170 (9)	0.416	170 (10)	169 (10)	170 (10)	0.421	170 (10)	169 (10)	170 (10)	0.243
Weight, kg, mean (SD)	79 (17)	80 (18)	78 (16)	0.309	79 (17)	80 (18)	79 (17)	0.597	79 (17)	79 (17)	79 (17)	0.677
BMI, kg/m^2^, mean (SD)	27.3 (5.8)	27.7 (6.6)	27.0 (4.9)	0.148	27.3 (5.8)	28.3 (6.5)	27.2 (5.7)	0.141	27.3 (6.0)	27.4 (5.0)	27.3 (5.8)	0.874
*Co-morbidities*, n (%)												
Co-morbidities	161 (20.5)	84 (22.3)	77 (18.9)	0.230	160 (20.6)	30 (37.0)	130 (18.7)	0.000	98 (17.9)	59 (29.5)	157 (21.0)	0.001
COPD	47 (6.0)	21 (5.6)	26 (6.4)	0.643	47 (6.0)	8 (9.9)	39 (5.6)	0.127	32 (5.8)	15 (7.5)	47 (6.3)	0.407
Respiratory	19 (2.4)	8 (2.1)	11 (2.7)	0.605	19 (2.4)	5 (6.2)	14 (2.0)	0.022	12 (2.2)	7 (3.5)	19 (2.5)	0.313
Liver cirrhosis	4 (0.5)	2 (0.5)	2 (0.5)	0.935	4 (0.5)	0 (0.0)	4 (0.6)	0.494	3 (0.5)	1 (0.5)	4 (0.5)	0.937
Chronic kidney failure	16 (2.0)	4 (1.1)	12 (2.9)	0.063	16 (2.1)	4 (4.9)	12 (1.7)	0.054	6 (1.1)	9 (4.5)	15 (2.0)	0.003
Heart failure	45 (5.7)	27 (7.2)	18 (4.4)	0.096	45 (5.8)	10 (12.3)	35 (5.0)	0.008	29 (5.3)	14 (7.0)	43 (5.7)	0.374
Neuro disease	12 (1.5)	8 (2.1)	4 (1.0)	0.191	12 (1.5)	2 (2.5)	10 (1.4)	0.476	11 (2.0)	1 (0.5)	12 (1.6)	0.146
**Pre-operative medical history**												
ASA physical status, n (%)	214 (27.4)	96 (25.5)	118 (29.1)	0.007	208 (26.8)	14 (17.5)	194 (27.9)	0.000	165 (30.1)	29 (14.6)	194 (26.0)	0.000
ASA I	395 (50.5)	178 (47.3)	217 (53.4)		395 (51.0)	33 (41.3)	362 (52.1)		292 (53.3)	90 (45.2)	382 (51.1)	
ASA II	395 (50.5)	178 (47.3)	217 (53.4)		153 (19.7)	28 (35.0)	125 (18.0)		85 (15.5)	68 (34.2)	153 (20.5)	
ASA III	154 (19.7)	87 (23.1)	67 (16.5)		18 (2.3)	5 (6.3)	13 (1.9)		6 (1.1)	11 (5.5)	17 (2.3)	
ASA IV	18 (2.3)	14 (3.7)	4 (1.0)		1 (0.1)	0 (0.0)	1 (0.1)		0 (0.0)	1 (0.5)	1 (0.1)	
ASA V	1 (0.1)	1 (0.3)	0 (0.0)		208 (26.8)	14 (17.5)	194 (27.9)		165 (30.1)	29 (14.6)	194 (26.0)	
Functional status, n (%)				0.004				0.000				0.006
Independent	708 (90.3)	327 (87.0)	381 (93.4)		702 (90.3)	67 (82.7)	635 (91.2)		506 (92.3)	168 (84.0)	674 (90.1)	
Partially dependent	62 (7.9)	38 (10.1)	24 (5.9)		62 (8.0)	12 (14.8)	50 (7.2)		33 (6.0)	27 (13.5)	60 (8.0)	
Totally dependent	13 (1.7)	11 (2.9)	2 (0.5)		12 (1.5)	2 (2.5)	10 (1.4)		8 (1.5)	5 (2.5)	13 (1.7)	
ARISCAT score, median (IQR)	215 (27.4)	102 (27.1)	113 (27.7)	0.000	16 (3; 26)	23 (11; 32)	16 (3; 24)	0.002	8 (3; 18)	31 (26; 37)	16 (3; 26)	0.000
Smoking, n (%)	40 (5.1)	23 (6.1)	17 (4.2)	0.859	214 (27.5)	22 (27.2)	192 (27.6)	0.442	165 (30.1)	44 (22.0)	209 (27.9)	0.029
Transfusion (< 24 h), n (%)	5 (0.6)	3 (0.8)	2 (0.5)	0.215	39 (5.0)	6 (7.4)	33 (4.7)	0.000	16 (2.9)	22 (11.0)	38 (5.1)	0.000
RBC transfusion (< 24 h)	28 (3.6)	16 (4.3)	12 (2.9)	0.589	5 (0.6)	1 (1.2)	4 (0.6)	0.722	1 (0.2)	4 (2.0)	5 (0.7)	0.007
Respiratory infection (<30d), n (%)	1 (0.1)	0 (0.0)	1 (0.2)	0.322	28 (3.6)	3 (3.7)	25 (3.6)	0.002	7 (1.3)	19 (9.5)	26 (3.5)	0.000
**Laboratory tests and vital signs**												
Pre-operative values												
SpO_2_, %, median (IQR)	97 (96; 99)	97 (96; 98)	97 (96; 99)	0.230	97 (96; 99)	97 (95; 98)	98 (96; 99)	0.002	98 (96; 99)	96 (94; 98)	97 (96; 99)	0.000
Hb, (g/dL), mean (SD)	13.8 (1.8)	13.8 (1.8)	13.9 (1.8)	0.540	13.8 (1.8)	13.7 (2.0)	13.8 (1.8)	0.442	14.0 (1.6)	13.3 (2.1)	13.8 (1.8)	0.000
WBC, (cell/mm^3^), mean (SD)	7879 (3497)	8199 (3097)	7568 (3825)	0.019	7891 (3503)	9261 (6168)	7721 (2978)	0.000	7696 (3362)	8438 (3845)	7905 (3518)	0.015
Creatinine, (mg/dL), mean (SD)	0.89 (0.69)	0.90 (0.86)	0.88 (0.49)	0.758	0.89 (0.69)	0.87 (0.28)	0.90 (0.73)	0.722	0.87 (0.59)	0.95 (0.91)	0.89 (0.70)	0.192
**Surgical characteristics**												
Condition, n (%)				0.318				0.140				0.000
Elective	717 (91.5)	338 (89.9)	379 (92.9)		712 (91.6)	73 (90.1)	639 (91.8)		513 (93.6)	172 (86.0)	685 (91.6)	
Urgency	50 (6.4)	28 (7.4)	22 (5.4)		49 (6.3)	4 (4.9)	45 (6.5)		31 (5.7)	16 (8.0)	47 (6.3)	
Emergency	17 (2.2)	10 (2.7)	7 (1.7)		16 (2.1)	4 (4.9)	12 (1.7)		4 (0.7)	12 (6.0)	16 (2.1)	
Planned duration, hours, n (%)				0.000				0.000				0.000
0	1 (0.1)	1 (0.3)	0 (0.0)		1 (0.1)	0 (0.0)	1 (0.1)					
≤ 2	432 (55.1)	186 (49.5)	246 (60.3)		426 (54.8)	36 (44.4)	390 (56.0)		378 (69.0)	25 (12.5)	403 (53.9)	
2–3	201 (25.6)	90 (23.9)	111 (27.2)		201 (25.9)	15 (18.5)	186 (26.7)		124 (22.6)	73 (36.5)	197 (26.3)	
> 3	150 (19.1)	99 (26.3)	51 (12.5)		149 (19.2)	30 (37.0)	119 (17.1)		46 (8.4)	102 (51.0)	148 (19.8)	
Antibiotic prophylaxis, n (%)	711 (90.9)	338 (90.1)	373 (91.6)	0.462	705 (91.0)	74 (91.4)	631 (90.9)	0.897	500 (91.4)	184 (92.0)	684 (91.6)	0.796

*P* value refers to the between-groups with Fisher-Freeman-Halton Exact test, Mann Whitney u-test, or Kruskal Wallis test, as appropriate. *N* Number, *IQR* Interquartile range, *SD* Standard deviation, *h* Hours, *d* Days, *PPC* Postoperative pulmonary complications, *COPD* Chronic obstructive pulmonary disease, *ASA* American society of anesthesiologists, *RBC* Blood red cells, *SpO*_*2*_ Blood oxygen saturation, *Hb* Hemoglobin, *WBC* White blood cells

### Ventilation variables and intraoperative characteristics

Most of the patients underwent elective surgical procedure (72%), with a median surgical duration of 95 min (1st-3rd interquartile range IQR = 60–160) and median anaesthetic time of 126 min (IQR = 90–192.8 min). The most common ventilation mode was volume-controlled ventilation (VCV) (Table 2). VCV was more commonly used in patients undergoing brain surgery. Median V_T_ was 510 ml (Interquartile range, IQR 475–575), thus resulting in 8 ml/kg predicted body weight (IQR = 7.3–9). Median PEEP level was 5 cmH_2_O (IQR 3–5), Ppeak was 18 cmH_2_O (IQR = 15–21) and driving pressure was 12 (IQR = 11–15) cmH_2_O (Table 2).

**Table 2:** Intra-Operative Characteristics of the Patients According to Subgroups

	All Patients	Brain	Spine	***p*** value	All patients	PPC	No PPC	***p*** value	All patients	ARISCAT < 26	ARISCAT ≥ 26	***p*** value
***n (%)***	784 (100.0)	376 (48.0)	408 (52.0)		777 (100.0)	81 (10.4)	696 (89.6)		748 (100.0)	548 (73.3)	200 (26.7)	
**Ventilation and vital signs**												
Ventilatory mode, n (%)				0.000				0.376				0.452
Volume controlled	494 (63.8)	259 (70.2)	235 (58.0)		488 (63.5)	50 (64.1)	438 (63.5)		467 (63.2)	341 (62.9)	126 (64.0)	
Pressure controlled	149 (19.3)	42 (11.4)	107 (26.4)		149 (19.4)	20 (25.6)	129 (18.7)		146 (19.8)	112 (20.7)	34 (17.3)	
Pressure support	3 (0.4)	2 (0.5)	1 (0.2)		3 (0.4)	0 (0.0)	3 (0.4)		3 (0.4)	1 (0.2)	2 (1.0)	
Spontaneous	64 (8.3)	21 (5.7)	43 (10.6)		64 (8.3)	4 (5.1)	60 (8.7)		60 (8.1)	42 (7.7)	18 (9.1)	
Other	64 (8.3)	45 (12.2)	19 (4.7)		64 (8.3)	4 (5.1)	60 (8.7)		63 (8.5)	46 (8.5)	17 (8.6)	
V_T_, ml, median (IQR)	510 (475; 575)	511 (475; 584)	506 (471; 562)	0.183	510 (475; 575)	500 (458; 560)	513 (475; 575)	0.096	510 (475; 572)	506 (475; 565)	525 (480; 590)	0.142
V_T_, (ml/kg PBW), median (IQR)	8.0 (7.3; 9.0)	8.2 (7.3; 9.1)	8.0 (7.2; 8.9)	0.150	8.0 (7.3; 9.0)	7.7 (7.0; 8.8)	8.1 (7.3; 9.0)	0.060	8.0 (7.3; 9.0)	8.0 (7.3; 9.0)	8.0 (7.3; 9.1)	0.420
PPeak, cmH_2_O, median (IQR)	18 (15; 21)	18 (15; 21)	18 (16; 21)	0.225	18 (15; 21)	18 (16; 21)	18 (15; 21)	0.183	18 (15; 21)	18 (15; 21)	18 (16; 21)	0.061
PPlateau, cmH_2_O, median (IQR)	16 (14; 19)	16 (14; 19)	16 (14; 18)	0.201	16 (14; 19)	17 (14; 19)	16 (14; 19)	0.150	16 (14; 19)	16 (14; 18)	17 (15; 19)	0.012
PEEP, cmH_2_O, median (IQR)	5.0 (3.0; 5.0)	5.0 (4.0; 5.0)	5.0 (3.0; 5.0)	0.669	5.0 (3.0; 5.0)	5.0 (4.0; 5.0)	5.0 (3.0; 5.0)	0.225	5.0 (3.0; 5.0)	5.0 (3.0; 5.0)	5.0 (3.3; 5.0)	0.156
DP, cmH_2_O, median (IQR)	12 (11; 15)	13 (11; 15)	12 (10; 16)	0.585	12 (11; 15)	13 (11; 15)	12 (11; 15)	0.201	12 (11; 15)	12 (11; 15)	14 (11; 17)	0.009
RR, bpm, mean (SD)	12.0 (1.5)	12.1 (1.5)	12.0 (1.4)	0.237	12.0 (1.5)	12.1 (1.7)	12.0 (1.4)	0.669	12.0 (1.4)	12.1 (1.3)	11.9 (1.7)	0.188
FiO_2_, %, median (IQR)	50 (43; 65)	50 (40; 60)	50 (44; 68)	0.021	50 (43; 64)	50 (46; 65)	50 (42; 63)	0.585	50 (43; 65)	50 (43; 70)	50 (45; 60)	0.143
SpO_2_, %, median (IQR)	99 (98; 100)	99 (99; 100)	99 (98; 100)	0.169	99 (98; 100)	99 (98; 100)	99 (98; 100)	0.237	99 (98; 100)	99 (99; 100)	99 (98; 100)	0.069
ETCO_2_, mmHg, mean (SD)	33 (4)	32 (4)	33 (5)	0.001	33 (4)	33 (4)	33 (5)	0.554	33 (4)	33 (4)	33 (5)	0.549
MP, J/min, median (IQR)	6.6 (4.9; 9.2)	6.9 (5.0; 10.3)	6.2 (4.8; 7.8)	0.058	6.6 (4.9; 9.2)	6.1 (4.8; 10.5)	6.6 (4.9; 9.1)	0.856	6.6 (4.9; 9.3)	6.6 (4.9; 8.6)	6.7 (5.1; 10.8)	0.230
MAP, mmHg, mean (SD)	80 (12)	79 (12)	80 (13)	0.083	79 (12)	78 (11)	80 (13)	0.021	79 (12)	79 (12)	80 (13)	0.212
Heart rate, bpm, mean (SD)	71 (12)	69 (12)	72 (12)	0.004	71 (12)	68 (12)	71 (12)	0.169	70 (12)	71 (12)	70 (13)	0.355
RM, n (%)	54 (6.9)	29 (7.8)	25 (6.1)	0.365	54 (6.9)	29 (7.8)	25 (6.1)	0.365	51 (6.8)	36 (6.6)	15 (7.5)	0.664
**Anesthesia characteristics**												
Opioids, n (%)												
No	2 (0.3)	0 (0.0)	2 (0.5)		2 (0.3)	0 (0.0)	2 (0.5)		90 (12.0)	65 (11.9)	25 (12.5)	
Yes	782 (99.7)	376 (100.0)	406 (99.5)	0.174	782 (99.7)	376 (100.0)	406 (99.5)	0.629	746 (99.7)	546 (99.6)	200 (100.0)	0.392
Opioids type, n (%)				0.000				0.055				0.836
Short acting	221 (28.2)	137 (36.4)	84 (20.6)		220 (28.3)	20 (24.7)	200 (28.7)		2 (0.3)	2 (0.4)	0 (0.0)	
Long acting	466 (59.4)	189 (50.3)	277 (67.9)		460 (59.2)	43 (53.1)	417 (59.9)		212 (28.3)	154 (28.1)	58 (29.0)	
Total fluids, ml, median (IQR)	1500 (1000; 2000)	1500 (1000; 2000)	1500 (1000; 2000)	0.022	1500 (1000; 2000)	1800 (1200; 2125)	1500 (1000; 2000)	0.001	1500 (1000; 2000)	1300 (1000; 2000)	2000 (1100; 3000)	0.000
Cristalloids	1175 (1000; 2000)	1200 (1000; 2000)	1000 (1000; 1500)	0.012	1200 (1000; 2000)	1500 (1000; 2050)	1000 (1000; 2000)	0.000	1200 (1000; 2000)	1000 (1000; 1500)	1725 (1000; 2475)	0.000
Colloids	0.0 (0.0; 500.0)	0.0 (0.0; 500.0)	0.0 (0.0; 500.0)	0.649	0.0 (0.0; 500.0)	0.0 (0.0; 500.0)	0.0 (0.0; 500.0)	0.719	0.0 (0.0; 500.0)	0.0 (0.0; 125.0)	0.0 (0.0; 500.0)	0.649

*P*-value refers to the between-groups difference with Fisher-Freeman-Halton Exact test, Mann Whitney u-test, or Kruskal Wallis test, as appropriate. *N* Number; *IQR* Interquartile range, *SD* Standard deviation, *PPC* Postoperative pulmonary complications, *PBW* Predicted body weight, *V*_*T*_ Tidal volume, *PPeak* Peak pressure, *PPlateau* Plateau pressure, *PEEP* Positive end-expiratory pressure, *DP* Driving pressure, *RR* Respiratory rate, *FiO*_*2*_ Fraction of inspired oxygen, *SpO*_*2*_ Blood oxygen saturation, *ETCO*_*2*_ End-tidal carbon dioxide, *MP* Mechanical power, *MAP* Mean arterial pressure, *HR* Heart rate, *RM* Recruitment maneuvers

**Fig. 1:**
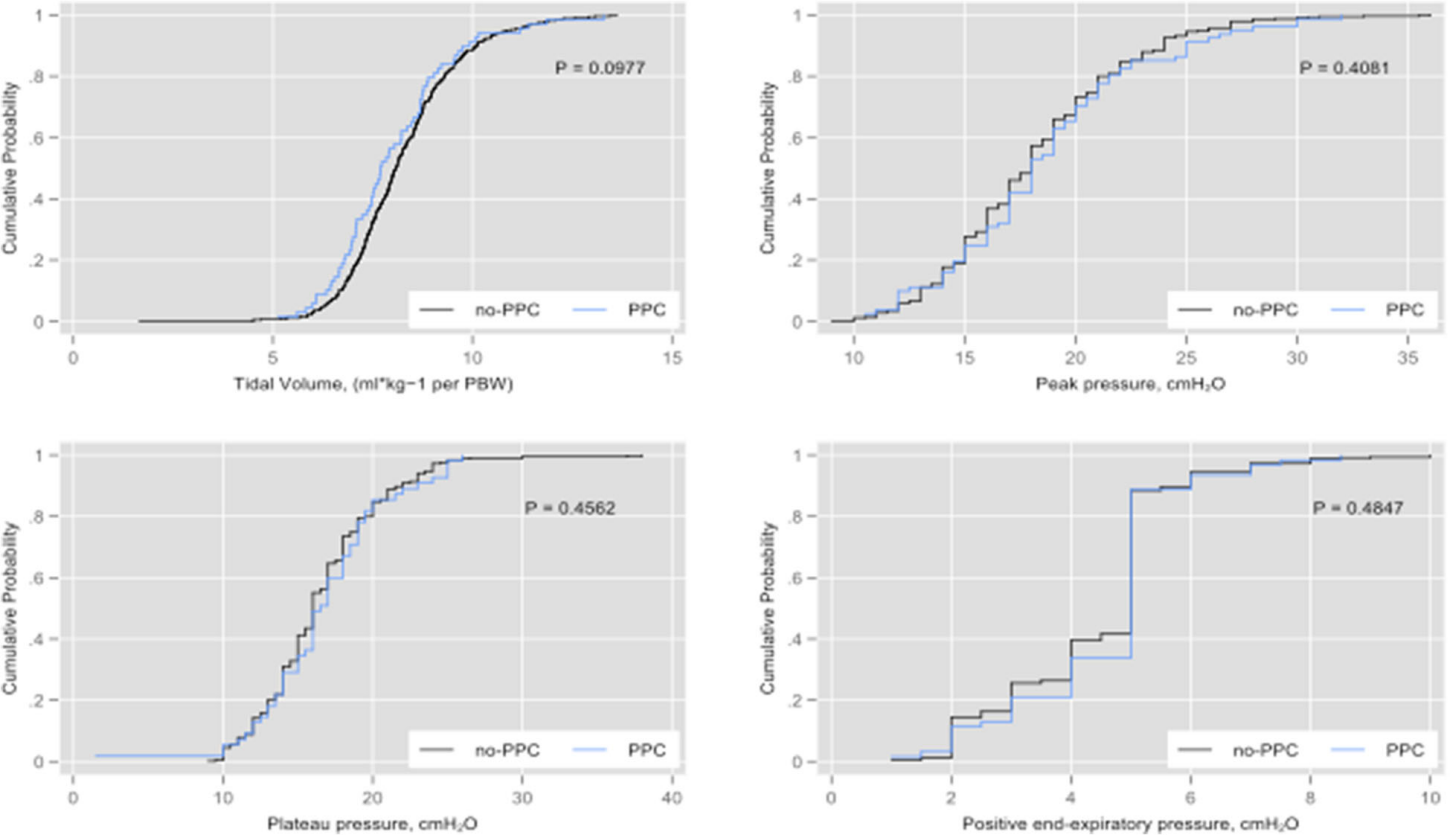
Ventilation parameters in patients who developed and who did not develop PPCs. Cumulative distribution of Tidal Volume (V_T_) (upper left panel); Cumulative distribution of peak pressure (Ppeak) (upper right panel); Cumulative distribution of plateau pressure (Pplat) (lower left panel); Cumulative frequency distribution of positive end expiratory pressure (PEEP) (lower right panel)

Routine RMs were performed in 54 patients (6.9%). Unplanned RMs occurred in 1.4% of cases. No statistical difference was found between the spine and brain surgery group or regarding the ventilator settings (Table 2, ESM Figure S1). EtCO_2_ values were significantly lower in the brain surgery group compared with the spine surgery group (*p* = 0.001). Patients who developed PPCs received a higher amount of fluids compared to those with no PPCs (Table 2), but no differences were found in the ventilator settings between the two groups (Fig. 1).

**Fig. 2:**
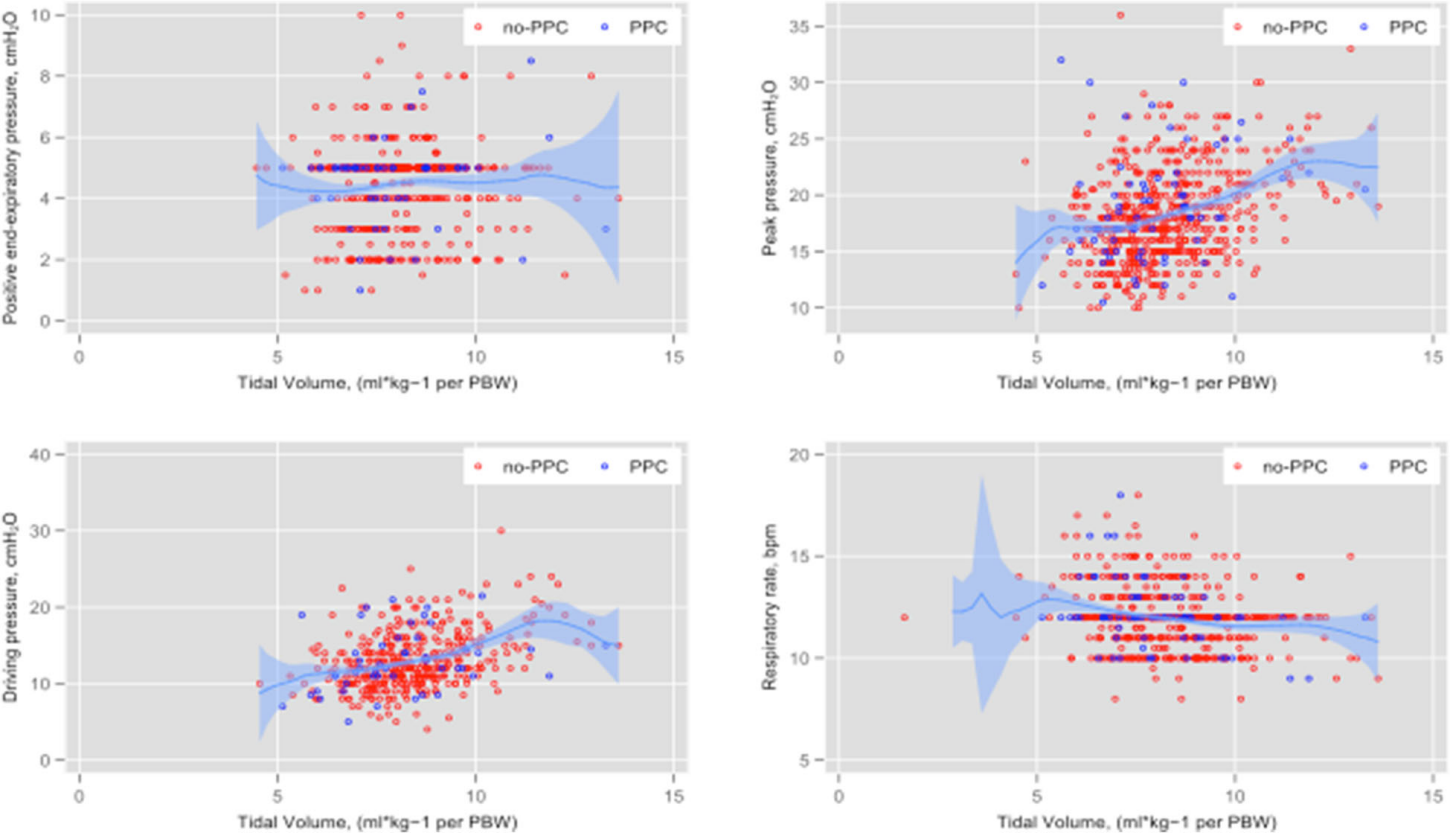
Combinations of ventilator settings in patients who developed or not developed PPCs. Scatterplots showing distribution of tidal volume with positive end expiratory pressure combinations (upper left panel); tidal volume with Peak pressure (upper right panel); tidal volume with Driving pressure (lower left panel); tidal volume and respiratory rate (lower right panel). Scatter and the fitted line for each of the bivariate plots are shown in blue

Scatter plots showing the combinations of V_T_ with PEEP, driving pressure, Ppeak, and respiratory rate in patients who developed versus patients who did not develop PPCs, between the spine and brain group, and in patients with low risk [ARISCAT < 26] vs. moderate-to-high risk [ARISCAT ≥26] are shown in Fig. 2, ESM Figures S2, S3.

### Occurrence of PPCs, intraoperative complications and outcomes

Among the 784 patients included in the analysis, 81 (10.4%) developed PPCs (Table 2). PPCs occurred mainly on day 3. No differences between the surgical groups were found as for probability of PPCs occurrence and hospital length of stay (ESM Figure S4).

Patients with ARISCAT≥26 showed an increased probability of PPCs occurrence compared to patients at lower risk (HR 2.50; 95% CI 1.61–3.58, *p* < 0.000), and of longer hospital length of stay (HR 0.81; 95% CI 0.69.0.97, *p* = 0.019) (ESM Figure S4).

Intraoperative episodes of hypotension and the need for vasoactive drugs during the procedure were frequent, especially in the spine group compared to the brain group (38.7% vs 31.2% for hypotension; *p* = 0.028 and 34.6% vs 27.7% for vasoactive drugs, *p* = 0.04, respectively) (Table 3). The incidence of desaturation was less frequent than hypotension or need of vasoactive drugs. No differences were found in terms of mortality or hospital length of stay in patients who developed and did not develop PPCs or the type of surgery. Patients with ARISCAT≥26 compared to those with ARISCAT< 26, had longer LOS and higher hospital mortality (Table 3).

**Table 3:** Outcomes According to Subgroups

	All Patients	Brain	Spine	***p*** value	All patients	PPC	No PPC	***p*** value	All patients	ARISCAT < 26	ARISCAT ≥ 26	***p*** value
***n (%)***	784 (100.0)	376 (48.0)	408 (52.0)		777 (100.0)	81 (10.4)	696 (89.6)		748 (100.0)	548 (73.3)	200 (26.7)	
**PPCs, n (%)**												
PPCs	81 (10.4)	46 (12.4)	35 (8.6)	0.085	777 (100.0)	81 (10.4)	696 (89.6)	0.000	80 (10.8)	43 (7.9)	37 (18.6)	0.000
Need of oxygen	69 (8.9)	38 (10.2)	31 (7.6)	0.202	81 (10.4)	81 (100.0)	0 (0.0)	0.000	68 (9.2)	39 (7.2)	29 (14.6)	0.002
Respiratory failure	14 (1.8)	8 (2.2)	6 (1.5)	0.478	69 (8.9)	69 (85.2)	0 (0.0)	0.000	14 (1.9)	6 (1.1)	8 (4.0)	0.010
NIV	9 (1.2)	4 (1.2)	5 (1.2)	0.963	14 (1.8)	14 (17.3)	0 (0.0)	0.000	9 (1.3)	7 (1.3)	2 (1.1)	0.801
ARDS	1 (0.1)	1 (0.3)	0 (0.0)	0.295	9 (1.2)	5 (6.3)	4 (0.6)	0.003	1 (0.1)	0 (0.0)	1 (0.5)	0.098
Pneumothorax	1 (0.1)	1 (0.3)	0 (0.0)	0.295	1 (0.1)	1 (1.2)	0 (0.0)	0.003	1 (0.1)	0 (0.0)	1 (0.5)	0.098
**Secondary outcomes, n (%)**												
Severe PPCs	19 (2.4)	13 (3.5)	6 (1.5)	0.068	19 (2.4)	19 (23.5)	0 (0.0)	0.000	19 (2.6)	6 (1.1)	13 (6.5)	0.000
Intra-operative complications	344 (43.9)	154 (41.1)	190 (46.6)	0.121	342 (44.1)	46 (56.8)	296 (42.6)	0.015	336 (44.9)	237 (43.2)	99 (49.5)	0.128
Desaturation	38 (4.9)	23 (6.1)	15 (3.7)	0.110	37 (4.8)	11 (13.6)	26 (3.7)	0.000	36 (4.8)	21 (3.8)	15 (7.5)	0.038
Unplanned RMs	25 (3.2)	15 (4.0)	10 (2.5)	0.220	24 (3.1)	5 (6.2)	19 (2.7)	0.091	22 (2.9)	12 (2.2)	10 (5.0)	0.043
Pressure reduction	25 (3.2)	11 (2.9)	14 (3.4)	0.692	25 (3.2)	3 (3.7)	22 (3.2)	0.795	22 (2.9)	17 (3.1)	5 (2.5)	0.666
Flow limitation	5 (0.6)	3 (0.8)	2 (0.5)	0.590	4 (0.5)	1 (1.3)	3 (0.4)	0.322	4 (0.5)	2 (0.4)	2 (1.0)	0.289
Hypotension	275 (35.1)	117 (31.2)	158 (38.7)	0.028	274 (35.3)	34 (42.0)	240 (34.5)	0.185	270 (36.1)	197 (35.9)	73 (36.5)	0.890
Vasopressors	245 (31.3)	104 (27.7)	141 (34.6)	0.040	244 (31.4)	37 (45.7)	207 (29.8)	0.004	242 (32.4)	168 (30.7)	74 (37.0)	0.101
New arrhythmias	9 (1.1)	6 (1.6)	3 (0.7)	0.257	9 (1.2)	0 (0.0)	9 (1.3)	0.303	9 (1.2)	5 (0.9)	4 (2.0)	0.227
Hospital LOS, days, median (IQR)	2 (1; 5)	2 (1; 5)	2 (1; 5)	0.993	2 (1; 5)	3 (1; 5)	2 (1; 5)	0.447	2 (1; 5)	2 (1; 5)	3 (1; 5)	0.033
Hospital mortality	5 (0.7)	4 (1.2)	1 (0.3)	0.145	5 (0.7)	1 (1.3)	4 (0.6)	0.500	5 (0.7)	1 (0.2)	4 (2.2)	0.006

*n* Number, *IQR* Interquartile range, *PPCs* Postoperative pulmonary complications, *NIV* Non-invasive ventilation, *ARDS* Acute respiratory distress syndrome, *LOS* Length of hospital stay, *RMs* Recruitment maneuvers, *ARISCAT* Assess respiratory risk in surgical patients in Catalonia

### Risk factors for PPCs

**Fig. 3:**
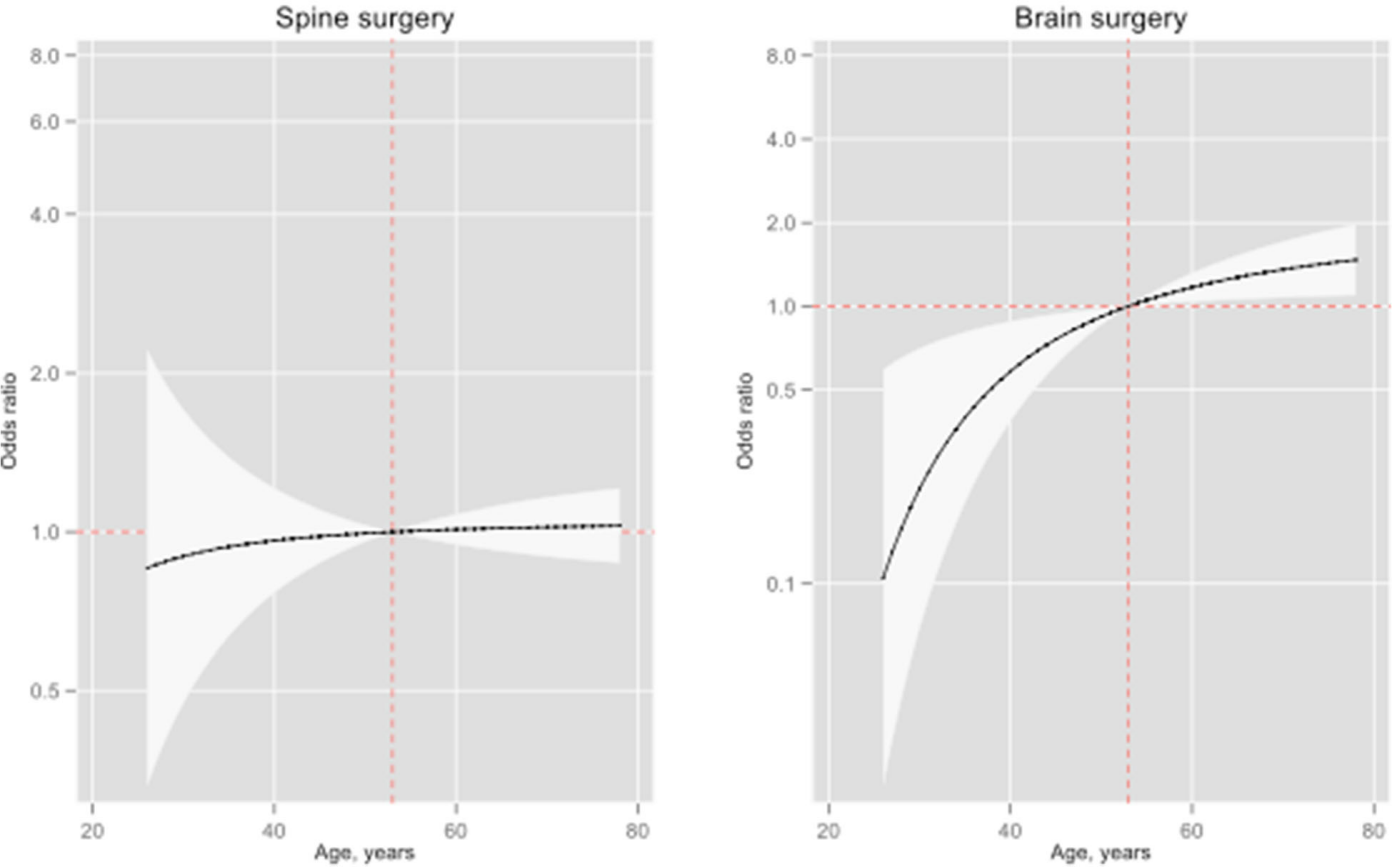
Interaction between type of neurosurgery and age continuous on PPC as outcome. Odds ratio (per 1-unit change in age) is depicted along the continuum of age (years) with its median (53 years) as reference point. Analysis adjusted by duration of anaesthesia, desaturation, and ARISCAT risk score. Indeed, the prognostic effect of age on PPC varies according to neurosurgery subpopulations, with no effect on the spine group, and a significant crescendo effect on the brain group as patient aged

Multivariable logistic regression was used to identify the predictors of PPCs. Duration of anaesthesia was independently associated for the development of PPCs. Analysing the predictors for type of neurosurgery, for age we found a significantly effect in the brain group (the omnibus *p*-value for the neurosurgery-age interaction was *p* = 0.031), but not in the spine group. (Table ESM S7, ESM Figure S5, Fig. 3). The effect of age on PPC in the brain group was significant at age above 62 (ESM Figure S5).

## Discussion

Our results show that: 1) Neurosurgical patients are ventilated with low V_T_ and low PEEP levels, while recruitment manoeuvres are seldom applied. No clinically significant differences exist between the intraoperative ventilator settings and the incidence of PPCs between the subgroups analysed, and in patients undergoing brain and spine surgery. ETCO_2_ levels are generally medium-low, especially in the brain surgery group; 2) PPCs are common, with similar incidence in the spine- and the brain surgical groups; 3) Intraoperative complications occur in a large number of patients (44% of the total population); of these, hypotension and the need for vasopressors are common; 4) Increasing age (for the brain group) and long surgical procedures are independently associated with development of PPCs.

To our knowledge, this is the first prospective observational study in neurosurgical patients specifically focusing on the prevalence of PPCs and the effects of intraoperative mechanical ventilation settings on PPCs development. Our study is a sub-analysis of the LAS VEGAS study [10], a large international observational study describing the ventilator settings and PPCs occurrence in the perioperative period across different countries, and can therefore be considered representative for the current clinical practice in this population.

### Ventilator strategies in patients undergoing neurosurgical interventions

Currently applied lung-protective ventilation strategies have shown to reduce PPCs [13, 14]. In patients undergoing spine surgery, the prone position has various effects on pulmonary function, including a decreased dynamic lung compliance and increased peak inspiratory pressure [13]; however, no large observational studies or randomized controlled trials are available regarding protective ventilator settings and their effect on PPCs in the prone position in non-ARDS patients.

In brain injured patients, lung-protective ventilation could be deleterious [7]; in particular, possible high intra-thoracic pressures when using high PEEP levels and permissive hypercapnia can have detrimental effects on cerebral perfusion pressure (CPP) and intracranial pressure (ICP). Therefore, brain injury patients are traditionally ventilated with tidal volumes approximating 9 ml/kg of PBW [5]. However, recent studies suggest that high V_T_ is a risk factor for acute lung injury even in patients with neurological disorders [4]. Indeed, our results suggest that the use of low V_T_ is increasingly applied also in neurosurgical patients. Similarly, the application of PEEP in brain injured patients has been traditionally considered detrimental for ICP, by reducing venous outflow [15]. However, recent evidence demonstrates that PEEP application might not compromise ICP, provided that arterial blood pressure is preserved [16, 17].

In our cohort, neurosurgical patients were ventilated with low PEEP levels and no differences in PEEP levels were detected between the brain and spine groups. No data is available on the effects of RM in neurosurgical patients and their role within the intraoperative protective ventilation bundle remains unclear. In brain injured patients, RMs can have a dangerous effect on ICP by impairment of jugular blood outflow, and increase of intra-thoracic pressure with impediment of cerebral venous return to the right atrium [8]. Although pressure-control recruitment manoeuvres improve oxygenation without impairing ICP or CPP, there is still concern regarding their application in neurosurgical patients, and therefore are rarely performed [8]. Indeed, our results show that recruitment manoeuvres are seldom applied in neurosurgical patients.

To date, no clinical studies comparing pressure-controlled ventilation (PCV) and VCV in brain injured patients are published. In obese [18], ARDS [19], and thoracic patients [20], research suggests no difference in outcome between the modes of ventilation (PCV and VCV). In a trial [21] including patients undergoing spinal surgery, PCV decreased intraoperative surgical bleeding compared with the VCV group (*p* < 0.001), possibly by lowering peak inspiratory pressures. A recent randomized controlled trial during lumbar spine surgery demonstrated that hemodynamic variables and arterial blood gas results did not differ significantly between the VCV and PCV with volume guaranteed (PCV-VG) mode groups [13]. Also, a recent large observational study suggested that PCV is associated with increase of PPC compared to VCV [22]. This association is not confirmed by our results. In our cohort, patients undergoing spinal surgery were more frequently ventilated with VCV than the brain injured group. However, despite the pathophysiological differences of prone vs supine ventilation, we did not find any other differences in the ventilator settings between the two groups.

In our cohort, ETCO_2_ levels were generally medium-low, with significantly lower values in the brain surgery group compared to the spinal surgery group. This result suggests that patients undergoing brain surgery are more likely to be hyperventilated. This is most likely out of concern for potential increased intracranial pressure.

Although the subgroup with ARISCAT ≥26 shows higher values of driving pressure and plateau pressure (plateau pressure (17 vs 16 cmH_2_O, *p* = 0.012), and higher driving pressure (14 vs 12 cmH_2_O; *p* = 0.009), these values still remain within the recommended ranges for protective ventilation [22, 23]. In general, in the whole population, a low total energy was applied to the respiratory system [23] (median mechanical power (6.2 J/min)), with values which remain far from the threshold of 12 J/min suggested as increased risk of lung injury [23].

### Post- operative pulmonary complications

Clinical studies suggest that the application of protective ventilation can reduce PPCs [24, 25], with high V_T_ identified as an independent predictor of PPCs development [26, 27]. Trials in obese [27] and non-obese [28] patients undergoing abdominal surgery demonstrated that the intraoperative application of high level of PEEP and RMs did not reduce PPCs, when compared with lower PEEP level without RMs.

In our neurosurgical population, 10.3% of patients developed PPCs, similar to the results from the whole population of the LAS VEGAS [8]. No clinically significant differences exist in the incidence of PPCs when comparing the different intraoperative ventilator settings in the subgroups analysed.

Patients who developed PPCs had worse preoperative conditions (age, ARISCAT score, ASA status), longer duration of anaesthesia (thus suggesting a more complicated surgical procedure), intraoperative complications (in particular hypotension) and the administration of higher volumes of fluid. This latter point is of extreme importance as cerebral and spinal perfusion pressures are generally maintained by the administration of vasopressors and a large amount of fluids; however, a positive fluid balance can increase the risk for pulmonary damage and complications [28]. Finally, increasing age in the brain surgical group was associated with PPCs occurrence, thus making preoperative assessment extremely important in the management of this group of patients in order to optimize hospital resources and empathetically begin discussions with patients and their carers.

### Intraoperative complications and outcomes

In our cohort, intraoperative complications occurred in a large number of patients (44% of our total population). Moreover, we found an increased prevalence of intraoperative hemodynamic deterioration as compared to respiratory impairment in the intraoperative settings. According to our results, patients undergoing spine surgery have commonly episodes of intraoperative hypotension requiring the use of vasoactive drugs, probably related to the effects of prone position on cardiac function, including a decreased cardiac index [13].

Our results suggest that in neurosurgical patients, the most common intraoperative complications are related to hemodynamic rather than respiratory function. The fact that hypotension and hemodynamic impairment are common might suggest that limited levels of PEEP could be beneficial in this type of patients by having less negative impact on hemodynamic. These results are in accordance with recently published literature [24, 29], suggesting that the use of high PEEP can negatively impact the hemodynamic system, thus challenging the traditional concept of “open lung approach”, and avoiding repeated alveolar collapse and expansion and keeping the lung partially at rest [30].

### Limitations

Several limitations need to be mentioned. First, the manuscript derives from a secondary analysis from the LAS VEGAS study. Thus, the results represent an observation of associations and do not allow to draw causality conclusions, considering that there exist unaccounted confounding factors.

Second, this is an unplanned secondary analysis from the main study, and even though we built a meticulous statistical model, there could still be confounding factors affecting our results.

Third, as the design of the original study focused on intraoperative settings and variables in the general population, limited information was available regarding specific perioperative data in neurosurgical patients, in particular on the use neuro-monitoring and type of brain and spine surgery.

## Conclusions

The main findings of this study are that MV settings in neurosurgical patients are characterized by low V_T_ and low PEEP with seldom use of RMs. PPCs are frequent in this population and not associated with intraoperative ventilator setting. Further studies are warranted to assess the effect of ventilation strategies on the outcome of this cohort of patients.

## Supplementary information

**Additional file 1.**

## Data Availability

The dataset used and analysed during the current study are available from the corresponding author on reasonable request.

## Abbreviations

V_T_: Tidal volume
Pplat: Plateau pressure
PEEP: Positive end-expiratory pressure
RM: Recruitment manoeuvres
PBW: Predicted body weight
PPCs: Post-operative pulmonary complications
ESM: Electronic supplemental material
Ppeak: Peak pressure
RR: Respiratory rate
FiO_2_: Fraction of inspired oxygen
ETCO_2_: End-tidal carbon dioxide
SpO_2_: Peripheral saturation of oxygen
MP: Mechanical power
ASA: American Society of Anaesthesiologists
ARISCAT: Assess Respiratory Risk in Surgical Patients in Catalonia
VCV: Volume-controlled ventilation
IQR: Interquartile range
SD: Standard deviation
PCV: Pressure-controlled ventilation
LOS: Length of stay
CPP: Cerebral perfusion pressure
ICP: Intracranial pressure
