# Supplementary material for: Intraoperative ventilator settings and their association with postoperative pulmonary complications in neurosurgical patients: post-hoc analysis of LAS VEGAS study
Source: BMC Anesthesiol. 2020 Apr 2;20:73. doi: 10.1186/s12871-020-00988-x (PMC7114790; doi:10.1186/s12871-020-00988-x)
Supplement: Supplementary file 1 — Additional file 1. [file 12871_2020_988_MOESM1_ESM.docx]

**Supplemental digital content to**

**“Intraoperative ventilator settings and their association with postoperative pulmonary complications in neurosurgical patients “**

**Contents**

Page 3 Table S1. STROBE Statement checklist

Page 6 List of LAS VEGAS study Network Collaborators

Page 13 Table S2. a, b. Full list of participating centres

Page 17 Table S3. Hospital characteristics of participating centres

Page 18 Table S4. Definition of recruitment manoeuvres

Page 18 Table S5. Definition of postoperative pulmonary complications

Page 19 Table S6. Definition of intraoperative complications

Page 20 Table S7. Univariate and multivariate logistic regression

Page 23 Figure FC. Flow-chart of patient’s inclusion criteria

Page 24 Figure S1. Settings of different hourly intraoperative factors over time.

Page 26 Figure S2. Combinations of ventilator settings in patients undergoing brain or spine surgery.

Page 28 Figure S3. Combinations of ventilator settings in patients with ARISCAT<26 and ARISCAT ≥26.

Page 30 Figure S4. Kaplan Meier plots

Page 32 Figure S5, Interaction between type of neurosurgery and age continuous on PPC as outcome

| Table S1. STROBE Statement checklist | | | |
| --- | --- | --- | --- |
|  | Item No. | Recommendation | Page  No. |
| **Title and abstract** | 1 | (*a*) Indicate the study’s design with a commonly used term in the title or the abstract | 1 |
|  |  | (*b*) Provide in the abstract an informative and balanced summary of what was done and what was found | 3 |
| Introduction | | | |
| Background/rationale | 2 | Explain the scientific background and rationale for the investigation being reported | 4 |
| Objectives | 3 | State specific objectives, including any pre–specified hypotheses | 4 |
| Methods | | | |
| Study design | 4 | Present key elements of study design early in the paper | 5 |
| Setting | 5 | Describe the setting, locations, and relevant dates, including periods of recruitment, exposure, follow-up, and data collection | 5 |
| Participants | 6 | (*a*) *Cohort study*—Give the eligibility criteria, and the sources and methods of selection of participants. Describe methods of follow-up | 5 |
|  |  | (*b*) *Cohort study*—For matched studies, give matching criteria and number of exposed and unexposed | NA |
| Variables | 7 | Clearly define all outcomes, exposures, predictors, potential confounders, and effect modifiers. Give diagnostic criteria, if applicable | 5 |
| Data sources/ measurement | 8* | For each variable of interest, give sources of data and details of methods of assessment (measurement). Describe comparability of assessment methods if there is more than one group | 6 |
| Bias | 9 | Describe any efforts to address potential sources of bias | 6 |
| Study size | 10 | Explain how the study size was arrived at | 6 |

Continued on next page

| Quantitative variables | 11 | Explain how quantitative variables were handled in the analyses. If applicable, describe which groupings were chosen and why | 6 |
| --- | --- | --- | --- |
| Statistical methods | 12 | (*a*) Describe all statistical methods, including those used to control for confounding | 7 |
|  |  | (*b*) Describe any methods used to examine subgroups and interactions | 7 |
|  |  | (*c*) Explain how missing data were addressed | 7 |
|  |  | (*d*) *Cohort study*—If applicable, explain how loss to follow-up was addressed | 7 |
|  |  | (*e*) Describe any sensitivity analyses | 7 |
| Participants | 13* | (a) Report numbers of individuals at each stage of study—e.g. numbers potentially eligible, examined for eligibility, confirmed eligible, included in the study, completing follow-up, and analysed | 6 |
|  |  | (b) Give reasons for non-participation at each stage | 6 |
|  |  | (c) Consider use of a flow diagram | 6 |
| Descriptive data | 14* | (a) Give characteristics of study participants (e.g. demographic, clinical, social) and information on exposures and potential confounders | 6 |
|  |  | (b) Indicate number of participants with missing data for each variable of interest | NA |
|  |  | (c) *Cohort study*—Summarise follow-up time (e.g., average and total amount) | NA |
| Outcome data | 15* | *Cohort study*—Report numbers of outcome events or summary measures over time | *7* |
| Main results | 16 | (*a*) Give unadjusted estimates and, if applicable, confounder-adjusted estimates and their precision (e.g., 95% confidence interval). Make clear which confounders were adjusted for and why they were included | 8-9 |
|  |  | (*b*) Report category boundaries when continuous variables were categorized | 8-9 |
|  |  | (*c*) If relevant, consider translating estimates of relative risk into absolute risk for a meaningful time period | 8-9 |

Continued on next page

| Other analyses | 17 | Report other analyses done - e.g. analyses of subgroups and interactions, and sensitivity analyses | NA |
| --- | --- | --- | --- |
| **Discussion** | | | |
| Key results | 18 | Summarise key results with reference to study objectives | 9,10 |
| Limitations | 19 | Discuss limitations of the study, taking into account sources of potential bias or imprecision. Discuss both direction and magnitude of any potential bias | 15 |
| Interpretation | 20 | Give a cautious overall interpretation of results considering objectives, limitations, multiplicity of analyses, results from similar studies, and other relevant evidence | 10-14 |
| Generalizability | 21 | Discuss the generalizability (external validity) of the study results | 11-14 |
| Other information | |  |  |
| Funding | 22 | Give the source of funding and the role of the funders for the present study and, if applicable, for the original study on which the present article is based | 2 |

**Table S2 a. List of LAS VEGAS study Network Collaborators**

Austria

***LKH Graz, Graz:*** Wolfgang Kroell, Helfried Metzler, Gerd Struber, Thomas Wegscheider

***AKH Linz, Linz*:** Hans Gombotz

***Medical University Vienna*:** Michael Hiesmayr, Werner Schmid, Bernhard Urbanek

Belgium

***UCL - Cliniques Universitaires Saint Luc Brussels*:** David Kahn, Mona Momeni, Audrey Pospiech, Fernande Lois, Patrice Forget, Irina Grosu

***Universitary Hospital Brussels (UZ Brussel)*:** Jan Poelaert, Veerle van Mossevelde, Marie-Claire van Malderen

***Het Ziekenhuis Oost Limburg (ZOL), Genk*:** Dimitri Dylst, Jeroen van Melkebeek, Maud Beran

***Ghent University Hospital, Gent*:** Stefan de Hert, Luc De Baerdemaeker, Bjorn Heyse, Jurgen Van Limmen, Piet Wyffels, Tom Jacobs, Nathalie Roels, Ann De Bruyne

***Maria Middelares, Gent*:** Stijn van de Velde

***European Society of Anaesthesiology, Brussels:*** *Brigitte Leva, Sandrine Damster, Benoit Plichon*

Bosnia and Herzegovina

***General Hospital “prim Dr Abdulah Nakas” Sarajevo*:** Marina Juros-Zovko, Dejana  Djonoviċ- Omanoviċ

Croatia

***General Hospital Cakovec, Cakovec*:** Selma Pernar

***General Hospital Karlovac, Karlovac*:** Josip Zunic, Petar Miskovic, Antonio Zilic

***University Clinical Hospital Osijek, Osijek*:** Slavica Kvolik, Dubravka Ivic, Darija Azenic-Venzera, Sonja Skiljic, Hrvoje Vinkovic, Ivana Oputric

***University Hospital Rijeka, Rijeka*:** Kazimir Juricic, Vedran Frkovic

***General Hospital Dr J Bencevic, Slavonski Brod*:** Jasminka Kopic, Ivan Mirkovic

***University Hospital Center Split, Split*:** Nenad Karanovic, Mladen Carev, Natasa Dropulic

***University Hospital Merkur, Zagreb*:** Jadranka Pavicic Saric, Gorjana Erceg, Matea Bogdanovic Dvorscak

***University Hospital Sveti Duh, Zagreb*:** Branka Mazul-Sunko, Anna Marija Pavicic, Tanja Goranovic

***University Hospital, Medical school, “Sestre milosrdnice” (Sister of Charity), Zagreb*:** Branka Maldini, Tomislav Radocaj, Zeljka Gavranovic, Inga Mladic-Batinica, Mirna Sehovic

Czech Republic

***University Hospital Brno, Brno*:** Petr Stourac, Hana Harazim, Olga Smekalova, Martina Kosinova, Tomas Kolacek, Kamil Hudacek, Michal Drab

***University Hospital Hradec Kralove, Hradec Kralove*:** Jan Brujevic, Katerina Vitkova, Katerina Jirmanova

***University Hospital Ostrava, Ostrava*:** Ivana Volfova, Paula Dzurnakova, Katarina Liskova

***Nemocnice Znojmo, Znojmo*:** Radovan Dudas, Radek Filipsky

Egypt

***El Sahel Teaching hospital, Cairo*:** Samir el Kafrawy

***Kasr Al-Ainy Medical School, Cairo University*:** Hisham Hosny Abdelwahab, Tarek Metwally, Ahmed Abdel-Razek

***Beni Sueif University Hospital, Giza*:** Ahmed Mostafa El-Shaarawy, Wael Fathy Hasan, Ahmed Gouda Ahmed

***Fayoum University Hospital, Giza*:** Hany Yassin, Mohamed Magdy, Mahdy Abdelhady

***Suis medical Insurance Hospital, Suis*:** Mohamed Mahran

Estonia

***North Estonia Medical Center, Tallinn*:** Eiko Herodes, Peeter Kivik, Juri Oganjan, Annika Aun

***Tartu University Hospital, Tartu*:** Alar Sormus, Kaili Sarapuu, Merilin Mall, Juri Karjagin

France

***University Hospital of Clermont-Ferrand, Clermont-Ferrand*:** Emmanuel Futier, Antoine Petit, Adeline Gerard

***Institut Hospitalier Franco-Britannique, Levallois-Perret*:** Emmanuel Marret, Marc Solier

***Saint Eloi University Hospital, Montpellier*:** Samir Jaber, Albert Prades

Germany

***Fachkrankenhaus Coswig, Coswig*:** Jens Krassler, Simone Merzky

***University Hospital Carl Gustav Carus, Dresden*:** Marcel Gama de Abreu, Christopher Uhlig,

Thomas Kiss, Anette Bundy, Thomas Bluth, Andreas Gueldner, Peter Spieth, Martin Scharffenberg, Denny Tran Thiem, Thea Koch

***Duesseldorf University Hospital, Heinrich-Heine University*:** Tanja Treschan, Maximilian Schaefer,

Bea Bastin, Johann Geib, Martin Weiss, Peter Kienbaum, Benedikt Pannen

***Diakoniekrankenhaus Friederikenstift, Hannover*:** Andre Gottschalk, Mirja Konrad, Diana Westerheide, Ben Schwertfeger

***University of Leipzig, Leipzig*:** Hermann Wrigge, Philipp Simon, Andreas Reske, Christian Nestler

Greece

***“Alexandra” general hospital of Athens, Athens*:** Dimitrios Valsamidis, Konstantinos Stroumpoulis

***General air force hospital, Athens*:** Georgios Anthopoulos, Antonis Andreaou, Dimitris Karapanos

***Aretaieion University Hospital, Athens*:** Kassiani Theodorak, Georgios Gkiokas, Marios-Konstantinos Ttasoulis

***Attikon University Hospital, Athens*:** Tatiana Sidiropoulou, Foteini Zafeiropoulou, Panagiota Florou, Aggeliki Pandazi

***Ahepa University Hospital Thessaloniki, Thessaloniki*:** Georgia Tsaousi, Christos Nouris, Chryssa Pourzitaki,

Israel

***The Lady Davis Carmel Medical Center, Haifa*:** Dmitri Bystritski, Reuven Pizov, Arieh Eden

Italy

***Ospedale San. Paolo Bari, Bari*:** Caterina Valeria Pesce, Annamaria Campanile, Antonella Marrella

***University of Bari “Aldo Moro”, Bari*:** Salvatore Grasso, Michele De Michele

***Institute for Cancer Research and treatment, Candiolo, Turin*:** Francesco Bona, Gianmarco Giacoletto, Elena Sardo

***Azienda Ospedaliera per l’emergenza Cannizzaro, Catania*:** Luigi Giancarlo Vicari Sottosanti

***Ospedale Melegnano, Cernuso, Milano*:** Maurizio Solca

***Azienda Ospedaliera – Universitaria Sant’Anna, Ferrara*:** Carlo Alberto Volta, Savino Spadaro, Marco Verri, Riccardo Ragazzi, Roberto Zoppellari

***Ospedali Riuniti Di Foggia - University of Foggia, Foggia*:** Gilda Cinnella, Pasquale Raimondo, Daniela La Bella, Lucia Mirabella, Davide D'antini

***IRCCS AOU San Martino IST Hospital, University of Genoa, Genoa*:** Paolo Pelosi, Alexandre Molin, Iole Brunetti, Angelo Gratarola, Giulia Pellerano, Rosanna Sileo, Stefano Pezzato, Luca Montagnani

***IRCCS San Raffaele Scientific Institute, Milano*:** Laura Pasin, Giovanni Landoni, Alberto Zangrillo, Luigi Beretta, Ambra Licia Di Parma, Valentina Tarzia, Roberto Dossi, Marta Eugenia Sassone

***Istituto europeo di oncologia – ieo, Milano*:** Daniele Sances, Stefano Tredici, Gianluca Spano, Gianluca Castellani, Luigi Delunas, Sopio Peradze, Marco Venturino

***Ospedale Niguarda Ca'Granda Milano, Milano*:** Ines Arpino, Sara Sher

***Ospedale San Paolo - University of Milano, Milano*:** Concezione Tommasino, Francesca Rapido, Paola Morelli

***University of Naples “Federico II” Naples*:** Maria Vargas, Giuseppe Servillo

***Policlinico "P. Giaccone", Palermo*:** Andrea Cortegiani, Santi Maurizio Raineri, Francesca Montalto, Vincenzo Russotto, Antonino Giarratano

***Azienda Ospedaliero-Universitaria, Parma*:** Marco Baciarello, Michela Generali, Giorgia Cerati

***Santa Maria degli Angeli, Pordenone*:** Yigal Leykin

***Ospedale Misericordia e Dolce - Usl4 Prato, Prato*:** Filippo Bressan, Vittoria Bartolini, Lucia Zamidei

***University hospital of Sassari, Sassari*:** Luca Brazzi, Corrado Liperi, Gabriele Sales, Laura Pistidda

***Insubria University, Varese*:** Paolo Severgnini, Elisa Brugnoni, Giuseppe Musella, Alessandro Bacuzzi

Republic of Kosovo

***Distric hospital Gjakova, Gjakove*:** Dalip Muhardri

***University Clinical Center of Kosova, Prishtina*:** Agreta Gecaj-Gashi, Fatos Sada

***Regional Hospital ”Prim.Dr. Daut Mustafa”, Prizren*:** Adem Bytyqi

Lithuania

***Medical University Hospital, Hospital of Lithuanian University of Health Sciences, Kaunas*:** Aurika Karbonskiene, Ruta Aukstakalniene, Zivile Teberaite, Erika Salciute

***Vilnius University Hospital - Institute of Oncology, Vilnius*:** Renatas Tikuisis, Povilas Miliauskas

***Vilnius University Hospital - Santariskiu Clinics, Vilnius*:** Sipylaite Jurate, Egle Kontrimaviciute, Gabija Tomkute

Malta

***Mater Dei Hospital, Msida*:** John Xuereb, Maureen Bezzina, Francis Joseph Borg

Netherlands

***Academic Medical Centre, University of Amsterdam*:** Sabrine Hemmes, Marcus Schultz, Markus Hollmann, Irene Wiersma, Jan Binnekade, Lieuwe Bos

***VU University Medical Center, Amsterdam*:** Christa Boer, Anne Duvekot

***MC Haaglanden, Den Haag*:** Bas in  ‘t  Veld, Alice Werger, Paul Dennesen, Charlotte Severijns

***Westfriesgasthuis, Hoorn*:** Jasper De Jong, Jens Hering, Rienk van Beek

Norway

***Haukeland University Hospital, Bergen*:** Stefan Ivars, Ib Jammer

***Førde Central Hospital /Førde Sentral Sykehus, Førde*:** Alena Breidablik

***Martina Hansens Hospital, Gjettum*:** Katharina Skirstad Hodt, Frode Fjellanger, Manuel Vico Avalos

***Bærum Hospital, Vestre Viken, Rud*:** Jannicke Mellin-Olsen, Elisabeth Andersson

***Stavanger University Hospital, Stavanger*:** Amir Shafi-Kabiri

Panama

***Hospital Santo Tomás, Panama*:** Ruby Molina, Stanley Wutai, Erick Morais

Portugal

***Hospital do Espírito Santo - Évora, E.P.E, Évora.*:** Glória Tareco, Daniel Ferreira, Joana Amaral

***Centro Hospitalar de Lisboa Central, E.P.E, Lisboa.*:** Maria de Lurdes Goncalves Castro, Susana Cadilha, Sofia Appleton

***Centro Hospitalar de Lisboa Ocidental, E.P.E. Hospital de S. Francisco Xavier, Lisboa*:** Suzana Parente, Mariana Correia, Diogo Martins

***Santarem Hospital, Santarem*:** Angela Monteirosa, Ana Ricardo, Sara Rodrigues

Romania

***Spital Orasenesc, Bolintin Vale*:** Lucian Horhota

***Clinical Emergency Hospital of Bucharest, Bucharest*:** Ioana Marina Grintescu, Liliana Mirea, Ioana Cristina Grintescu

***Elias University Emergency Hospital, Bucharest*:** Dan Corneci, Silvius Negoita, Madalina Dutu, Ioana Popescu Garotescu

***Emergency Institute of Cardiovascular Diseases Inst. ''Prof. C. C. Iliescu'', Bucharest*:** Daniela Filipescu, Alexandru Bogdan Prodan

***Fundeni Clinical institute - Anaesthesia and Intensive Care, Bucharest*:** Gabriela Droc, Ruxandra Fota, Mihai Popescu

***Fundeni Clinical institute - Intensive Care Unit, Bucharest*:** Dana Tomescu, Ana Maria Petcu, Marian Irinel Tudoroiu

***Hospital Profesor D Gerota, Bucharest*:** Alida Moise, Catalin-Traian Guran

***Constanta County Emergency Hospital, Constanta*:** Iorel Gherghina, Dan Costea, Iulia Cindea

***University Emergency County Hospital Targu Mures, Targu Mures*:** Sanda-Maria Copotoiu, Ruxandra Copotoiu, Victoria Barsan, Zsolt Tolcser, Magda Riciu, Gheorghe Moldovan Septimiu, Mihaly Veres

Russia

***Krasnoyarsk State Medical University, Krasnoyarsk*:** Alexey Gritsan, Tatyana Kapkan, Galina Gritsan, Oleg Korolkov

***Burdenko Neurosurgery Institute, Moscow*:** Alexander Kulikov, Andrey Lubnin

***Moscow Regional Research Clinical Institute, Moscow*:** Alexey Ovezov, Pavel Prokoshev, Alexander Lugovoy, Natalia Anipchenko

***Municipal Clinical Hospital 7, Moscow*:** Andrey Babayants, Irina Komissarova, Karginova Zalina

***Reanimatology Research Institute n.a. Negovskij RAMS, Moscow*:** Valery Likhvantsev, Sergei Fedorov

Serbia

***Clinical Center of Vojvodina, Emergency Center, Novisad*:** Aleksandra Lazukic, Jasmina Pejakovic, Dunja Mihajlovic

Slovakia

***National Cancer Institute, Bratislava*:** Zuzana Kusnierikova, Maria Zelinkova

***F.D. Roosevelt teaching Hospital, Banská Bystrica*:** Katarina Bruncakova, Lenka Polakovicova

***Faculty Hospital Nové Zámky, Nové Zámky*:** Villiam Sobona

Slovenia

***Institute of Oncology Ljubljana, Ljubljana*:** Barbka Novak-Supe, Ana Pekle-Golez, Miroljub Jovanov, Branka Strazisar

***University Medical Centre Ljubljana, Ljubljana*:** Jasmina Markovic-Bozic, Vesna Novak-Jankovic, Minca Voje, Andriy Grynyuk, Ivan Kostadinov, Alenka Spindler-Vesel

Spain

***Hospital Sant Pau, Barcelona*:** Victoria Moral, Mari Carmen Unzueta, Carlos Puigbo, Josep Fava

***Hospital Universitari Germans Trias I Pujol, Barcelona*:** Jaume Canet, Enrique Moret, Mónica Rodriguez Nunez, Mar Sendra, Andrea Brunelli, Frederic Rodenas

***University of Navarra, Pamplona*:** Pablo Monedero, Francisco Hidalgo Martinez, Maria Jose Yepes Temino, Antonio Martínez Simon, Ana de Abajo Larriba

***Corporacion Sanitaria Parc Tauli, Sabadell*:** Alberto Lisi, Gisela Perez, Raquel Martinez

***Consorcio Hospital General Universitario de Valencia, Valencia*:** Manuel Granell, Jose Tatay Vivo, Cristina Saiz Ruiz, Jose Antonio de Andrés Ibañez

***Hospital Clinico Valencia, Valencia*:** Ernesto Pastor, Marina Soro, Carlos Ferrando, Mario Defez

***Hospital Universitario Rio Hortega, Valladolid*:** Cesar Aldecoa Alvares-Santullano, Rocio Perez, Jesus Rico

Sweden

***Central Hospital in Kristianstad*:** Monir Jawad, Yousif Saeed, Lars Gillberg

Turkey

***Ufuk University Hospital Ankara, Ankara*:** Zuleyha Kazak Bengisun, Baturay Kansu Kazbek

***Akdeniz University Hospital, Antalya*:** Nesil Coskunfirat, Neval Boztug, Suat Sanli, Murat Yilmaz, Necmiye Hadimioglu

***Istanbul University, Istanbul medical faculty, Istanbul*:** Nuzhet Mert Senturk, Emre Camci, Semra Kucukgoncu, Zerrin Sungur, Nukhet Sivrikoz

***Acibadem University, Istanbul*:** Serpil Ustalar Ozgen, Fevzi Toraman

***Maltepe University, Istanbul*:** Onur Selvi, Ozgur Senturk, Mine Yildiz

***Dokuz Eylül Universitesi Tip Fakültesi, Izmir*:** Bahar Kuvaki, Ferim Gunenc, Semih Kucukguclu, Şule Ozbilgin

***Şifa University Hospital, İzmir*:** Jale Maral, Seyda Canli

***Selcuk University faculty of medicine, Konya*:** Oguzhan Arun, Ali Saltali, Eyup Aydogan

***Fatih Sultan Mehmet Eğitim Ve Araştirma Hastanesi, Istanbul*:** Fatma Nur Akgun, Ceren Sanlikarip, Fatma Mine Karaman

Ukraine

***Institute Of Surgery And Transplantology, Kiev*:** Andriy Mazur

***Zaporizhzhia State Medical University, Zaporizhzhia*:** Sergiy Vorotyntsev

United Kingdom

SWARM Research Collaborative: for full list of SWARM contributors please see www.ukswarm.com

***Northern Devon Healthcare NHS Trust, Barnstaple*:** Guy Rousseau, Colin Barrett, Lucia Stancombe

***Golden Jubilee National Hospital, Clydebank, Scotland*:** Ben Shelley, Helen Scholes

***Darlington Memorial Hospital, County Durham and Darlington Foundation NHS Trust, Darlington*:** James Limb, Amir Rafi, Lisa Wayman, Jill Deane

***Royal Derby Hospital, Derby:*** David Rogerson, John Williams, Susan Yates, Elaine Rogers

***Dorset County Hospital, Dorchester*:** Mark Pulletz, Sarah Moreton, Stephanie Jones

***The Princess Alexandra NHS Hospital Trust, Essex*:** Suresh Venkatesh, Maudrian Burton, Lucy Brown, Cait Goodall

***Royal Devon and Exeter NHS Foundation Trust, Exeter*:** Matthew Rucklidge, Debbie Fuller, Maria Nadolski, Sandeep Kusre

***Hospital James Paget University Hospital NHS Foundation Trust, Great Yarmouth*:** Michael Lundberg, Lynn Everett, Helen Nutt

***Royal Surrey County Hospital NHS Foundation Trust, Guildford*:** Maka Zuleika, Peter Carvalho, Deborah Clements, Ben Creagh-Brown

***Kettering General Hospital NHS Foundation Trust, Kettering*:** Philip Watt, Parizade Raymode

***Barts Health NHS Trust, Royal London Hospital, London*:** Rupert Pearse, Otto Mohr, Ashok Raj, Thais Creary

***Newcastle Upon Tyne Hospitals NHS Trust The Freeman Hospital High Heaton, Newcastle upon Tyne*:** Ahmed Chishti, Andrea Bell, Charley Higham, Alistair Cain, Sarah Gibb, Stephen Mowat

***Derriford Hospital Plymouth Hospitals NHS Trust, Plymouth*:** Danielle Franklin, Claire West, Gary Minto, Nicholas Boyd

***Royal Hallamshire Hospital, Sheffield*:** Gary Mills, Emily Calton, Rachel Walker, Felicity Mackenzie, Branwen Ellison, Helen Roberts

***Mid Staffordshire NHS, Stafford*:** Moses Chikungwa, Clare Jackson

***Musgrove Park Hospital, Taunton*:** Andrew Donovan, Jayne Foot, Elizabeth Homan

***South Devon Healthcare NHS Foundation Trust /Torbay Hospital, Torquay, Torbay*:** Jane Montgomery, David Portch, Pauline Mercer, Janet Palme

***Royal Cornwall Hospital, Truro*:** Jonathan Paddle, Anna Fouracres, Amanda Datson, Alyson Andrew, Leanne Welch

***Mid Yorkshire Hospitals NHS Trust; Pinderfields Hospital, Wakefield*:** Alastair Rose, Sandeep Varma, Karen Simeson

***Sandwell and West Birmingham NHS Trust, West Bromich*:** Mrutyunjaya Rambhatla, Jaysimha Susarla, Sudhakar Marri, Krishnan Kodaganallur, Ashok Das, Shivarajan Algarsamy, Julie Colley

***York Teaching Hospitals NHS Foundation Trust, York*:** Simon Davies, Margaret Szewczyk, Thomas Smith

United States

***University of Colorado School of Medicine/University of Colorado Hospital, Aurora*:** Ana Fernandez- Bustamante, Elizabeth Luzier, Angela Almagro

***Massachusetts General Hospital, Boston*:** Marcos Vidal Melo, Luiz Fernando, Demet Sulemanji

***Mayo Clinic, Rochester*:** Juraj Sprung, Toby Weingarten, Daryl Kor, Federica Scavonetto, Yeo Tze

| **Table S2 b. Full list of participating centres** | | | |
| --- | --- | --- | --- |
| **Country** | **City** | **Institution** | **# Patients** |
| Austria | Graz | LKH Graz | 51 |
| Austria | Linz | AKH Linz | 40 |
| Austria | Vienna | Medical University Vienna | 34 |
| Belgium | Brussels | UCL - Cliniques Universitaires Saint Luc Brussels | 119 |
| Belgium | Brussels | Universitary Hospital Brussels (UZ Brussel) | 27 |
| Belgium | Genk | Het ZOL (Ziekenhuis Oost Limburg) | 65 |
| Belgium | Gent | Ghent University Hospital | 154 |
| Belgium | Gent | Maria Middelares Gent | 10 |
| Bosnia and Herzegovina | Sarajevo | General Hospital “prim Dr Abdulah Nakas” Sarajevo | 48 |
| Croatia | Cakovec | General Hospital Čakovec | 40 |
| Croatia | Karlovac | General Hospital Karlovac | 28 |
| Croatia | Osijek | University Clinical Hospital Osijek | 125 |
| Croatia | RIJEKA | University Hospital Rijeka | 53 |
| Croatia | Slavonski Brod | General Hospital Dr J Bencevic | 46 |
| Croatia | Split | University Hospital Center Split | 96 |
| Croatia | Zagreb | University Hospital Merkur | 48 |
| Croatia | Zagreb | University Hospital Sveti Duh | 59 |
| Croatia | Zagreb | University Hospital, Medical school, “Sestre milosrdnice” (Sister of Charity) | 85 |
| Czech Republic | Brno | University Hospital Brno | 149 |
| Czech Republic | Hradec Kralove | University Hospital Hradec Kralove | 82 |
| Czech Republic | Ostrava | University Hospital Ostrava | 102 |
| Czech Republic | Znojmo | Nemocnice Znojmo, p.o. | 62 |
| Egypt | Cairo | El Sahel Teaching hospital | 62 |
| Egypt | Cairo | Kasr Al-Ainy Medical School, Cairo University | 38 |
| Egypt | Giza | Beni Sueif University Hospital | 42 |
| Egypt | Giza | Fayoum University Hospital | 49 |
| Egypt | Suis | Suis medical Insurance Hospital | 48 |
| Estonia | Tallinn | North Estonia Medical Center | 164 |
| Estonia | Tartu | Tartu University Hospital | 80 |
| France | Clermont-Ferrand | Estaing Hospital, University Hospital of Clermont-Ferrand | 30 |
| France | Levallois-Perret | Institut Hospitalier Franco-Britannique | 21 |
| France | Montpellier | Saint Eloi University Hospital, Montpellier | 140 |
| Germany | Coswig | Fachkrankenhaus Coswig Gmbh, centre for pneumology and thoracic surgery | 10 |
| Germany | Dresden | University Hospital Carl Gustav Carus | 219 |
| Germany | Duesseldorf | Duesseldorf University Hospital, Heinrich-Heine University | 129 |
| Germany | Hannover | Diakoniekrankenhaus Friederikenstift | 133 |
| Germany | Leipzig | University of Leipzig | 71 |
| Greece | Athens | “Alexandra” general hospital of Athens | 43 |
| Greece | Athens | 251 General air force hospital | 41 |
| Greece | Athens | Aretaieion University Hospital | 41 |
| Greece | Athens | Attikon University Hospital | 54 |
| Greece | Thessaloniki | Ahepa University Hospital Thessaloniki | 50 |
| Israel | Haifa | The Lady Davis Carmel Medical Center | 40 |
| Italy | Bari | Ospedale San. Paolo Bari | 35 |
| Italy | Bari | University of Bari “Aldo Moro” | 81 |
| Italy | Candiolo (Turin) | Institute for Cancer Research and treatment | 28 |
| Italy | Catania | Azienda Ospedaliera per l’emergenza Cannizzaro | 39 |
| Italy | Cernuso (Milano) | Ospedale Melegnano | 5 |
| Italy | Ferrara | Azienda Ospedaliera – Universitaria Sant’Anna (Ferrara) | 88 |
| Italy | Foggia | Ospedali Riuniti Di Foggia - University of Foggia | 85 |
| Italy | Genoa | IRCCS San Martino AOU IST Hospital, University of Genoa | 173 |
| Italy | Milano | IRCCS San Raffaele Scientific Institute | 156 |
| Italy | Milano | Istituto europeo di oncologia - ieo | 131 |
| Italy | Milano | Ospedale Niguarda Ca'Granda Milano | 19 |
| Italy | Milano | Ospedale San Paolo - University of Milano | 46 |
| Italy | Naples | University of Naples “Federico II” | 57 |
| Italy | Palermo | Policlinico "P. Giaccone" | 92 |
| Italy | Parma | Azienda Ospedaliero-Universitaria | 81 |
| Italy | Pordenone | Santa Maria degli Angeli | 18 |
| Italy | Prato | Ospedale Misericordia e Dolce - Usl4 Prato | 41 |
| Italy | Sassari | University hospital of Sassari | 54 |
| Italy | Varese | Insubria University | 125 |
| Lithuania | Kaunas | Kaunas Medical University Hospital, Hospital of Lithuanian University of Health Sciences | 160 |
| Lithuania | Vilnius | Vilnius University Hospital - Institute of Oncology | 108 |
| Lithuania | Vilnius | Vilnius University Hospital - Santariskiu Clinics | 104 |
| Malta | Msida | Mater Dei Hospital | 27 |
| Netherlands | Amsterdam | Academic Medical Center, University of Amsterdam | 183 |
| Netherlands | Amsterdam | VU University Medical Center | 104 |
| Netherlands | Den Haag | MC Haaglanden | 94 |
| Netherlands | Hoorn | Westfriesgasthuis | 104 |
| Norway | Bergen | Haukeland University Hospital | 106 |
| Norway | Førde | Førde Central Hospital /Førde Sentral Sykehus | 58 |
| Norway | Gjettum | Martina Hansens Hospital | 57 |
| Norway | Rud | Bærum Hospital, Vestre Viken | 57 |
| Norway | Stavanger | Stavanger University Hospital | 70 |
| Panama | Panama | Hospital Santo Tomás | 40 |
| Portugal | Évora | Hospital do Espírito Santo - Évora, EPE. | 55 |
| Portugal | Lisboa | Centro Hospitalar de Lisboa Central, EPE | 49 |
| Portugal | Lisboa | Centro Hospitalar de Lisboa Ocidental, EPE | 41 |
| Portugal | Santarem | Santarem Hospital | 44 |
| Republic of Kosovo | Gjakovë | Distric hospital Gjakova | 9 |
| Republic of Kosovo | Prishtina | University Clinical Center of Kosova | 92 |
| Republic of Kosovo | Prizren | Regional Hospital ”Prim.Dr. Daut Mustafa” | 39 |
| Romania | Bolintin Vale | Spital orasenesc Bolintin Vale | 8 |
| Romania | Bucharest | Clinical Emergency Hospital of Bucharest | 58 |
| Romania | Bucharest | Elias University Emergency Hospital | 53 |
| Romania | Bucharest | Emergency Institute of Cardiovascular Diseases Inst. ''Prof. C. C. Iliescu'' | 6 |
| Romania | Bucharest | Fundeni Clinical institute - Anaesthesia and Intensive Care | 43 |
| Romania | Bucharest | Fundeni Clinical institute - Intensive Care Unit | 40 |
| Romania | Bucharest | Hospital Profesor D Gerota | 21 |
| Romania | Constanta | Constanta County Emergency Hospital | 73 |
| Romania | Targu Mures | University Emergency County Hospital Targu Mures | 138 |
| Russia | Krasnoyarsk | Krasnoyarsk State Medical University | 52 |
| Russia | Moscow | Burdenko Neurosurgery Institute | 60 |
| Russia | Moscow | Moscow Regional Research Clinical Institute | 59 |
| Russia | Moscow | Municipal Clinical Hospital 7 | 49 |
| Russia | Moscow | Reanimatology Research Institute n.a. Negovskij RAMS | 61 |
| Serbia | Novisad | Clinical Center of Vojvodina, Emergency Center | 42 |
| Slovakia | Bratislava | National Cancer Institute | 18 |
| Slovakia | Banská Bystrica | F.D.Roosevelt teaching Hospital | 64 |
| Slovakia | Nové Zámky | Faculty Hospital Nove Zamky | 47 |
| Slovenia | Ljubljana | Institute of Oncology Ljubljana | 64 |
| Slovenia | Ljubljana | University Medical Centre Ljubljana | 108 |
| Spain | Barcelona | Hospital Sant Pau | 73 |
| Spain | Barcelona | Hospital Universitari Germans Trias I Pujol | 103 |
| Spain | Pamplona | University of Navarra | 90 |
| Spain | Sabadell | Corporacion Sanitaria Parc Tauli | 66 |
| Spain | Valencia | Consorcio Hospital General Universitario de Valencia | 61 |
| Spain | Valencia | Hospital Clinico Valencia | 62 |
| Spain | Valladolid | Hospital Universitario Rio Hortega | 82 |
| Sweden | Kristianstad | Central Hospital in Kristianstad | 83 |
| Turkey | Ankara | Ufuk University Hospital Ankara | 30 |
| Turkey | Antalya | Akdeniz University Hospital | 165 |
| Turkey | Istanbul | Istanbul university, Istanbul medical faculty | 81 |
| Turkey | Istanbul | Acibadem University | 25 |
| Turkey | Istanbul | Maltepe University | 37 |
| Turkey | Izmir | Dokuz Eylül Universitesi Tip Fakültesi | 161 |
| Turkey | Izmir | Şifa University Hospital / İzmir | 74 |
| Turkey | Konya | Selcuk University faculty of medicine | 138 |
| Turkey | Istanbul | Fatih Sultan Mehmet Eğitim Ve Araştirma Hastanesi | 53 |
| Ukraine | Kiev | Institute Of Surgery And Transplantology | 17 |
| Ukraine | Zaporizhzhia | Zaporizhzhia State Medical University | 18 |
| United Kingdom | Barnstaple | Northern Devon Healthcare NHS Trust | 69 |
| United Kingdom | Clydebank, Scotland | Golden Jubilee National Hospital | 67 |
| United Kingdom | Darlington | Darlington Memorial Hospital, County Durham and Darlington Foundation NHS Trust | 54 |
| United Kingdom | Derby | Royal Derby Hospital | 59 |
| United Kingdom | Dorchester | Dorset County Hospital | 63 |
| United Kingdom | Essex | The Princess Alexandra NHS Hospital Trust | 71 |
| United Kingdom | Exeter | Royal Devon and Exeter NHS Foundation Trust | 123 |
| United Kingdom | Great Yarmouth | Hospital James Paget University Hospital NHS Foundation Trust | 43 |
| United Kingdom | Guildford | Royal Surrey County Hospital NHS Foundation Trust | 70 |
| United Kingdom | Kettering | Kettering General Hospital NHS Foundation Trust | 40 |
| United Kingdom | London | Barts Health NHS Trust, Royal London Hospital | 104 |
| United Kingdom | Newcastle upon Tyne | Newcastle Upon Tyne Hospitals NHS Trust The Freeman Hospital High Heaton | 125 |
| United Kingdom | Plymouth | Derriford Hospital Plymouth Hospitals NHS Trust | 179 |
| United Kingdom | Sheffield | Royal Hallamshire Hospital | 103 |
| United Kingdom | Stafford | Mid Staffordshire NHS | 28 |
| United Kingdom | Taunton | Musgrove Park Hospital | 50 |
| United Kingdom | Torquay, Torbay | South Devon Healthcare NHS Foundation Trust /Torbay Hospital, | 83 |
| United Kingdom | Truro | Royal Cornwall Hospital | 81 |
| United Kingdom | Wakefield | Mid Yorkshire Hospitals NHS Trust; Pinderfields Hospital | 65 |
| United Kingdom | West Bromich | Sandwell and West Birmingham NHS Trust | 127 |
| United Kingdom | York | York Teaching Hospitals NHS Foundation Trust | 81 |
| United States | Aurora, Colorado | University of Colorado School of Medicine/University of Colorado Hospital | 56 |
| United States | Boston | Massachusetts General Hospital, Boston | 120 |
| United States | Rochester | Mayo Clinic | 200 |

| **Table S3. Hospital characteristics of participating centres** | | |
| --- | --- | --- |
|  | | Centres responded (N = 145) |
| Community hospital | | 33 |
| Teaching hospital | | 108 |
| Number of hospital beds | | 600 [400 – 960] |
| Number of ICU beds | | 20 [11 – 35] |
| Number of operating theatres | | 15 [9 -22] |
| Staff (anaesthesiologists) | | 25 [12 – 40] |
| Invasive mechanical ventilation for surgery per week | | 103 [60 – 200] |
| Surgical procedures | | |
| Abdominal surgery | 135 (93%) |  |
| Bariatric surgery | 66 (46%) |  |
| Cardiothoracic surgery | 71 (49%) |  |
| Endocrine surgery | 88 (61%) |  |
| Eye surgery | 97 (67%) |  |
| General surgery | 132 (91%) |  |
| Gynaecological surgery | 126 (87%) |  |
| Interventional neuroradiology | 56 (39%) |  |
| Neurosurgery | 82 (57%) |  |
| Oral and maxillofacial surgery | 96 (62%) |  |
| Orthopaedic surgery | 121 (83%) |  |
| Plastic surgery | 91 (63%) |  |
| Surgical oncology | 107 (74%) |  |
| Trauma surgery | 111 (77%) |  |
| Urology | 121 (83%) |  |
| Vascular surgery | 109 (75%) |  |
| Data are presented as median [QR] or N (%). One participating centre did not respond; ICU: intensive care unit | | |

| **Table S4. Definition of recruitment manoeuvres** |  |
| --- | --- |
| **Type of recruitment manoeuvre** | **Definition** |
| Bag squeezing | Sustained manual hyperinflation using balloon/bag |
| Ventilator | Transient stepwise increase in positive end expiratory pressure (PEEP) at constant tidal volume or transient stepwise increase in tidal volume at constant PEEP or inspiratory holds (continuous positive airway pressure (CPAP) applied for a fixed time) or any combination of the previous three |
| Rescue recruitment manoeuvre | Bag-squeezing or ventilator recruitment manoeuvre, and “unplanned recruitment manoeuvre” reported as intraoperative complication |

| **Table S5. Definition of post-operative pulmonary complications (PPCs). Severe PPCs were defined as complications 2-5** | |
| --- | --- |
| **Single PPC** | **Definition** |
| 1.Unplanned need for oxygen therapy | Supplemental O_2_ therapy administered to correct hypoxemia, defined as SpO2 < 90% in room air or PaO_2_ < 60 mmHg. This excludes oxygen administration given as a part of standard care, such as routine oxygen administration at the arrival in the PACU |
| 2.Respiratory failure | SpO_2_ < 90% in room air or PaO_2_ < 60 mmHg with oxygen therapy, or need for non-invasive mechanical ventilation |
| 3.Mechanical ventilation | Need for new invasive ventilation after surgery, or unexpected prolonged invasive ventilation after discharge from the operating room |
| 4.Acute Distress Respiratory Syndrome | According to the Berlin definition |
| Pneumonia | Lung infiltrates at the chest X-ray or Computed Tomography, plus at least two among the following three criteria: fever > 38°C (100.4 °F), leucocytosis or leukopenia (WBC count > 12000 or <4000 cells/mm^3^), purulent secretions |
| 5.Pneumothorax | Air in the pleural space without blood presence, as confirmed by chest X-ray |

| **Table S6. Definition of intraoperative complications** | |
| --- | --- |
| Desaturation | Oxygen saturation, SpO_2_ < 92% |
| Need for rescue recruitment manoeuvre | Ventilation strategies aimed at restoring aeration of the lungs |
| Need for ventilatory pressure reduction | Ventilation strategies aimed at lowering peak and/or plateau pressure |
| New onset of expiratory flow limitation | End-expiration expiratory flow higher than zero at the visual analysis of the flow curve |
| Hypotension | Systolic arterial pressure < 90 mmHg for at least 3 minutes |
| Need for vasoactive drugs | Use of vasoactive drugs to correct hypotension as previously defined |
| Arrhythmia | Defined as any new onset of atrial fibrillation, sustained ventricular tachycardia, supraventricular tachycardia, or ventricular fibrillation |

| **Univariate models** | | | | |
| --- | --- | --- | --- | --- |
| **Variables** | **OR** | **95% CIs** | | **P-value** |
|  |  | **lower bound** | **upper bound** |  |
| Age, per 10 years | 1.345 | 1.174 | 1.539 | 0.000 |
| Gender | 1.238 | 0.780 | 1.964 | 0.364 |
| BMI, per 5 kg/height^2^ | 1.147 | 0.943 | 1.395 | 0.169 |
| ASA, per 1 unit | 1.818 | 1.302 | 2.539 | 0.000 |
| Smoker | 0.979 | 0.610 | 1.570 | 0.929 |
| COPD | 1.846 | 0.784 | 4.348 | 0.161 |
| Duration of anaesthesia, per 120 min | 1.452 | 1.196 | 1.762 | 0.000 |
| Respiratory infection <30 days | 1.032 | 0.338 | 3.153 | 0.955 |
| Total fluids, per 1000 ml | 1.264 | 1.088 | 1.469 | 0.002 |
|  | | | | |
| **Intraoperative complications** | | | | |
| Desaturation | 4.043 | 1.303 | 12.550 | 0.016 |
| Hypotension | 1.371 | 0.904 | 2.080 | 0.037 |
| Use of vasoactive drugs | 1.982 | 0.922 | 4.261 | 0.080 |
| Rescue Recruitment Manoeuvres Ventilator | 2.337 | 0.661 | 8.262 | 0.188 |
|  | | | | |
| **Ventilatory parameters** | | | | |
| Driving pressure, per 5 cmH_2_O | 1.014 | 0.713 | 1.441 | 0.940 |
| PEEP, per 1 cmH_2_O | 1.067 | 0.849 | 1.340 | 0.580 |
| Tidal Volume mL/kg PBW | 0.959 | 0.905 | 1.016 | 0.159 |
| Peak Pressure | 1.139 | 0.821 | 1.580 | 0.437 |
| FiO_2_ | 1.008 | 0.994 | 1.023 | 0.257 |
| Flow limitation | 2.991 | 0.377 | 23.709 | 0.300 |
| Routine Recruitment Manoeuvres Ventilator | 1.383 | 0.573 | 3.338 | 0.471 |
| Type of neurosurgery | 1.500 | 0.871 | 2.585 | 0.144 |
| ARISCAT risk score, per 1 unit | 1.037 | 1.020 | 1.053 | 0.000 |
| ARISCAT risk score ≥ 26 | 2.661 | 1.674 | 4.230 | 0.000 |
|  |  |  |  |  |
| **Multivariate model** | | | | |
| **Predictors** | **OR** | **95% CIs** | | **P-value** |
|  |  | **lower bound** | **upper bound** |  |
| Duration of anaesthesia, per 120 min | 1.295 | 1.067 | 1.572 | 0.009 |
| Desaturation | 3.115 | 0.933 | 10.407 | 0.065 |
| Hypotension | 3.014 | 0.912 | 10.313 | 0.085 |
| ARISCAT risk score ≥ 26 | 1.675 | 0.978 | 2.869 | 0.060 |
| Type of Neurosurgical procedure | 2.759 | 1.216 | 6.260 | 0.115 |
| Age | 0.244 | 0.000 | 1197.249 | 0.745 |
| Age according to type of Neurosurgical procedure | 0.000 | 0.000 | 0.189 | 0.031 |

**Table S7.** Univariate and multivariate logistic regression for the correlation of general intraoperative complications and ventilator parameters and the development of post pulmonary complications. Abbreviations: BMI, Body mass index; ASA, American Society of Anesthesiologists; COPD, chronic obstructive pulmonary disease; PEEP, positive end expiratory pressure; PBW, predicted body weight; FiO_2_, Fraction of inspired oxygen; OR, odds ratio; CI, confidence interval.

ESM. Flowchart

**
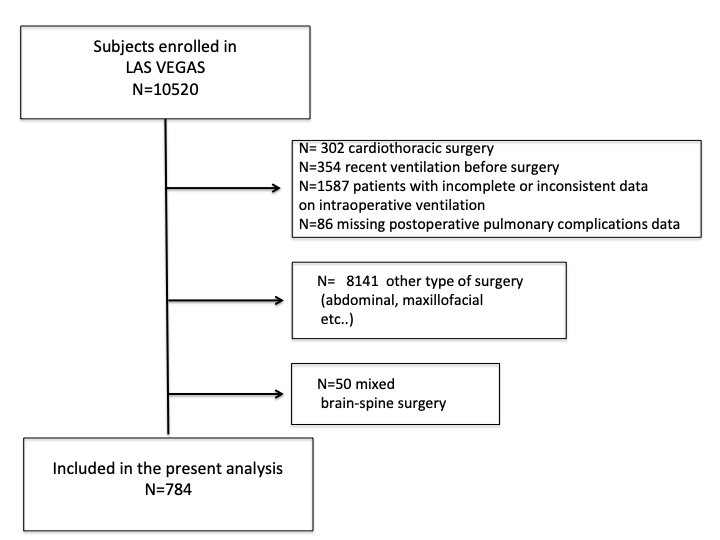
**

**Figure S1**. Settings of different hourly intraoperative factors over time. A, Positive End Expiratory Pressure (PEEP); B, inspired Fraction of Oxygen (FiO_2_); C, Oxygen saturation (SpO_2_); D, mean arterial pressure (MAP); E, Peak pressure (Ppeak); F, Tidal Volume (V_T_); G, carbon dioxide (CO_2_); H, respiratory rate (RR). T_0_ represents the induction of general anaesthesia. Continuous line represents the spine group and dashed line represents the brain group.

**
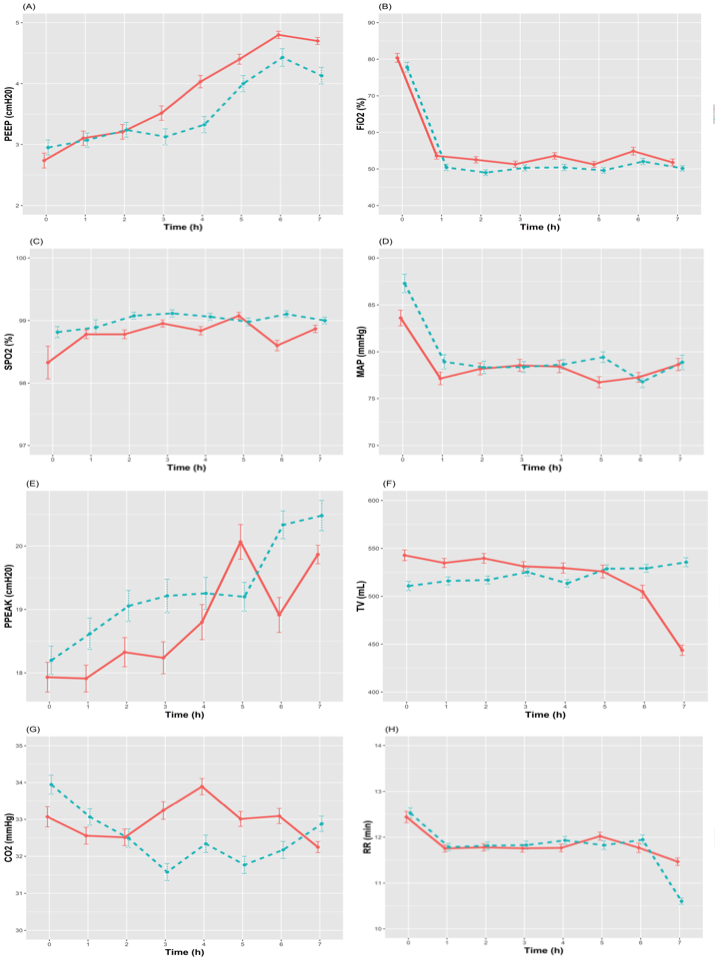
**

**Figure S2. Combinations of ventilator settings in patients undergoing brain or spine surgery.** Scatterplots showing distribution of tidal volume with positive end expiratory pressure combinations (upper left panel); tidal volume with Peak pressure (upper right panel); tidal volume with Driving pressure (lower left panel); tidal volume and respiratory rate (lower right panel). Scatter and the fitted line for each of the bivariate plots are shown in blue

**
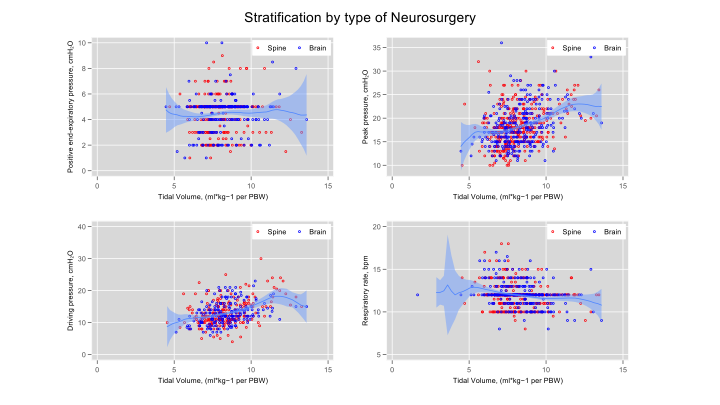
**

**Figure S3.Combinations of ventilator settings in patients with ARISCAT<26 and ARISCAT** ≥26**.** Scatterplots showing distribution of tidal volume with positive end expiratory pressure combinations (upper left panel); tidal volume with Peak pressure (upper right panel); tidal volume with Driving pressure (lower left panel); tidal volume and respiratory rate (lower right panel). Scatter and the fitted line for each of the bivariate plots are shown in blue

**
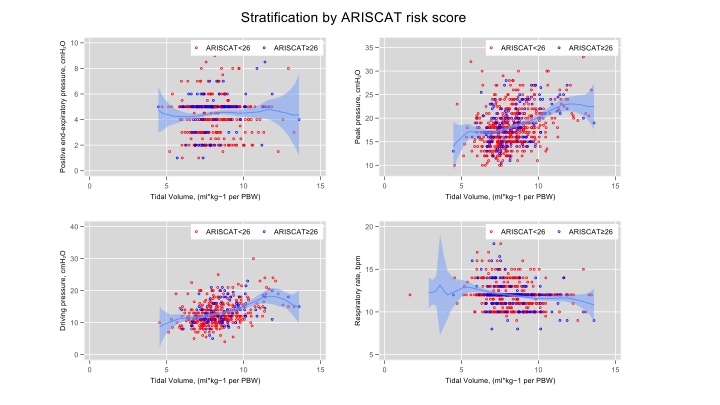
**

**Figure S4.** Kaplan-Meier plot for probability of hospital discharge and the occurrence of PPC over time for neurosurgical patients, stratified according to the type of surgery, and ARISCAT score. Test of equality Log Rank (Mantel-Cox) shows no statistical difference on length of stay and PPC occurrence between the type of surgery, but significant difference between patients with ARISCAT < 26 and ≥ 26.


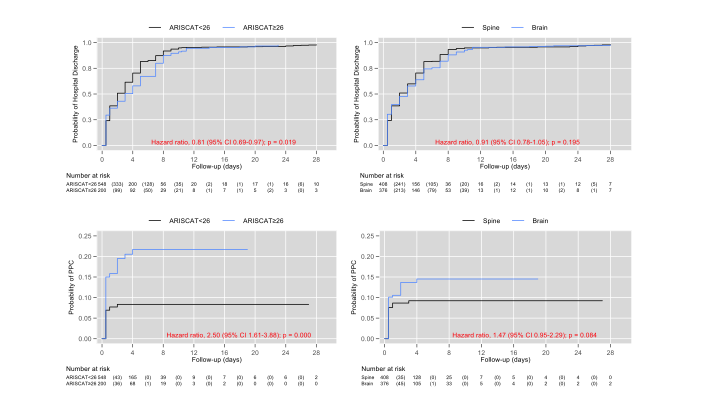


**Figure S5.** Interaction between type of neurosurgery and age continuous on PPC as outcome. This figure shows the delta log-odds - Brain minus spine surgery - per 1-unit change in age. Analysis adjusted by duration of anesthesia, desaturation, and ARISCAT risk score. By looking at the portion of the lower CI bound above the 0-line, we can conclude that the effect of age on PPC only becomes significant at age above 62. No effect between the two types of surgery was demonstrated below age 62.
